# Tracking the spread of a naturally occurring *Leishmania* (*L.*) *infantum* mutant: a qPCR-based investigation in strains and clinical samples

**DOI:** 10.1186/s13071-026-07571-2

**Published:** 2026-08-11

**Authors:** Marne Coimbra das Chagas, Ana Nilce Silveira Elkhoury, Samantha Valadas, Bruna Dias das Chagas, Camilly Enes, Artur Augusto Velho Mendes Junior, Sandro Antonio Pereira, Gabriel Carvalho de Macedo, André Luiz Rodrigues Roque, Manuela da Silva Solcà, Claudia Brodskyn, Deborah Bittencourt Mothé, Juliana Mariotti Guerra, José Eduardo Tolezano, Luiz Fabio Batista, Marcia Dalastra Laurenti, Lisvane Paes-Vieira, Carlos Robello, Otacilio C. Moreira, Elisa Cupolillo, Mariana Côrtes Boité

**Affiliations:** 1 Laboratório de Pesquisa em Leishmanioses, Instituto Oswaldo Cruz, Fundação Oswaldo Cruz, Fiocruz, Rio de Janeiro, RJ Brazil; 2 Communicable Diseases and Environmental Determinants of Health (CDE), Neglected, Tropical and Vector Borne Diseases (VT), Pan American Health Organization, World Health Organization (PAHO/WHO), Rio de Janeiro, Brazil; 3 Laboratório de Pesquisa Clínica em Dermatozoonoses em Animais Domésticos-Instituto Nacional de Infectologia Evandro Chagas, Fundação Oswaldo Cruz, Fiocruz, Rio de Janeiro, RJ Brazil; 4 Laboratório de Biologia de Tripanosomatídeos, Instituto Oswaldo Cruz, Fundação Oswaldo Cruz, Fiocruz, Rio de Janeiro, RJ Brazil; 5 Universidade Católica Dom Bosco, Campo Grande, MS Brazil; 6 Instituto Gonçalo Moniz, Fundação Oswaldo Cruz, Fiocruz, Salvador, BA Brazil; 7 Escola de Medicina Veterinária e Zootecnia, Universidade Federal da Bahia, Salvador, BA Brazil; 8 Departamento de Patologia, Faculdade de Medicina Veterinária e Zootecnia, Universidade de São Paulo, São Paulo, SP Brazil; 9 Centro de Parasitologia e Micologia, Instituto Adolfo Lutz, São Paulo, SP Brazil; 10 Laboratory of Pathology of Infectious Diseases, Medical School, São Paulo University, São Paulo, SP Brazil; 11 Laboratorio de Interacciones Hospedero-Patógeno-UBM, Institut Pasteur de Montevideo, Montevideo, Uruguay; 12 Laboratory of Molecular Virology and Parasitology, Instituto Oswaldo Cruz, Fundação Oswaldo Cruz, Fiocruz, Rio de Janeiro, Brazil

**Keywords:** *Leishmania*, *Leishmania infantum*, Genotypes, American visceral leishmaniasis, Deletion, Canine leishmaniasis

## Abstract

**Background:**

The co-circulation of Leishmania (*Leishmania*) *infantum* strains carrying a naturally occurring 12 kb genomic deletion (DEL) and the wild-type (NonDEL) parasites in the Americas raises important challenges for epidemiological surveillance, molecular diagnostics, and the interpretation of infection dynamics in visceral leishmaniasis (VL). In this study, we aimed to develop and apply a robust molecular approach to discriminate and quantify DEL and NonDEL genotypes and to assess their distribution, frequency, and patterns of co-infection across endemic regions.

**Methods:**

We established a qPCR-based genotyping strategy combining a constitutive genomic target, present in all *Leishmania (L.) infantum* strains, with a NonDEL-specific target, enabling simultaneous genotype discrimination and parasite quantification.

**Results:**

After validation using cultured parasites, the assay was applied to a large and diverse panel of strains isolates from humans and dogs preserved in a biobank, as well as clinical samples from dogs, cats, coatis, and sand flies across South and Central America. DEL parasites predominated (69.8%) and showed a wide geographic distribution in both strains and clinical samples, indicating their major contribution to transmission cycles. No significant association was observed between host type (human vs. canine) and genotype frequency. Notably, mixed-genotype infections were frequently detected, revealing within-host complexity not captured by conventional diagnostic methods. Quantification based on the constitutive target strongly correlated with kDNA-based parasite load estimates, supporting its simultaneous use for genotyping and quantification of L. (*L.*) *infantum* variants directly from clinical material.

**Conclusions:**

This approach thus provides new insights into parasite population structure and infection dynamics and supports improved molecular surveillance and interpretation of diagnostic and epidemiological data in VL.

**Graphical Abstract:**

**Figure:**
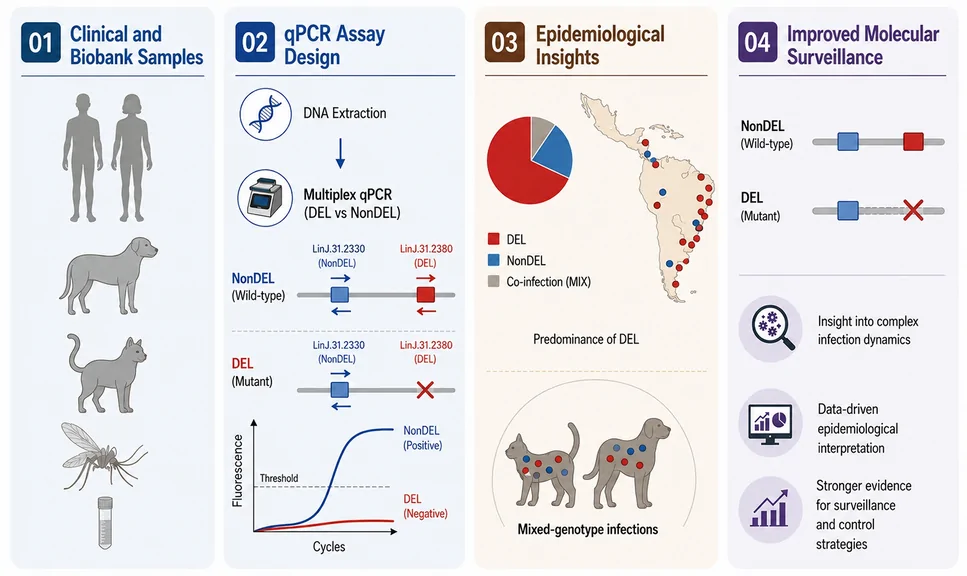

**Supplementary Information:**

The online version contains supplementary material available at https://doi.org/10.1186/s13071-026-07571-2.

## Background

*Leishmania* (*L.*) *infantum* is the agent of American visceral leishmaniasis (AVL), an important, although neglected, disease. The main urban reservoir is the domestic dog, which also can develop the disease, leading to canine visceral leishmaniasis (CVL) [1] or canine leishmaniasis (cL). The transmission cycle includes vector sandflies of various species, depending on the region. The main vector is *Lutzomyia longipalpis*, widely spread in South America [2], also documented in Central America and southern Mexico [3]. *Lutzomyia pseudolongipalpis* has been reported in Venezuela as a vector for the parasite [4], while *Lutzomyia evansi* was described as participating in the transmission of the parasite in Colombia and Venezuela [5, 6]. *Lutzomyia cruzi* is found in municipalities in the border areas between Bolivia and Brazil and between Bolivia and Argentina [6]. *Lutzomyia migonei* has also been described as a permissive vector for *L.* (*L*.) *infantum* [7] in Brazil and in Argentina [8].

Epidemiological changes of AVL are noteworthy, mainly, the occurrence of the disease, which continues to expand geographically, in both humans and dogs [9, 10]. The overall number of cases in the Americas has diminished in the recent years, but when countries are analyzed separately, Brazil harbors more than 90% of human AVL cases [11], and Paraguay and Argentina reveal an increase in the numbers [6]. Even though the absolute number of deaths lowered in the Americas, the lethality rate increased [11]. These modifications may be related to a complex overlapping of different factors. The adaptation of the vector sandfly species to the urban environment, for instance, is quite relevant in the disease expansion scenario. Another major player that may lead to modifications on the AVL scenario is the parasite itself. Studies have demonstrated that the parasites that circulate in the Americas belong to the same species present in Europe, Africa, and Asia, that arrived in the Americas during the last five centuries [12, 13]. The consequent bottleneck effect during such parasite importation, including the adaptation to novel vertebrate (native fauna) and invertebrate hosts (vector species that are not present in the Old World), possibly fostered genomic variation over the New World *L.* (*L*.) *infantum* population, which has been under investigation [13, 14]. As a consequence of comparative deep sequencing analysis efforts including genomes from different continents, a specific and relevant finding has been published: a 12 KB sub-chromosomal deletion, described exclusively among Brazilian strains of *L.* (*L.*) *infantum* [15, 16]. The deletion is homozygous, spanning across all four copies of tetrasomic chromosome 31 (chr31). It covers four open reading frames as follows: LinJ.31.2370 (ecto-3′-nucleotidase/nuclease), LinJ.31.2380 (ecto-3’-nucleotidase precursor), LinJ.31.2390 (helicase-like protein), and LinJ.31.2400 (3,2-trans-enoyl-CoA isomerase).

Some epidemiologically relevant phenotypes correlate with this parasite’s genomic trait. Our group recently described higher metacyclogenesis in vitro and in vivo (in *Lu. Longipalpis* infections) for deletion-carrying parasites (DEL), and an ability to infect the vertebrate host despite its susceptibility to the first cell line defense [17]. These findings suggest that DEL mutant strains could alter pathogenicity and lead to less symptomatic clinical outcome. Emphasizing the main urban reservoir, the domestic dog, this might represent a threat to surveillance and disease control since these animals could remain for longer periods undetected as a source of infection for the vector, contributing to keeping the endemicity in certain areas.

Notably, the deletion in *L.* (*L*.) *infantum* has been associated with reduced susceptibility to miltefosine (MIL) [15, 16], a key drug for treating both cutaneous and visceral leishmaniasis in several countries. In Brazil, however, due to the low efficacy observed during a VL clinical trial [16], MIL is currently approved only for treating human cutaneous leishmaniasis and cL. The use of MIL in dogs raises concerns for surveillance and control, as the same parasite population circulates between humans and dogs [18]. Moreover, dogs are often asymptomatic or present heterogeneous clinical manifestations that do not necessarily correlate with parasite load. While treatment can achieve clinical improvement [19], its impact on infectivity to vectors remains unclear [20], as does the role of treated dogs in maintaining transmission in urban settings, since current treatments for dogs do not provide parasitological cure.

In this scenario, the parasite’s genomic site of the deletion may represent a pivotal molecular marker for clinicians and surveillance to follow up the epidemiology of VL in the Americas. The currently known distribution of the *L.* (*L.*) *infantum* DEL and NonDEL genotypes is based exclusively on few strains (convenient samples) kept in a biological collection [14]. Based on these statements, the present work presented an expanded and detailed mapping of the occurrence of genotypes in clinical samples, avoiding the potential bias of culture-selection. To achieve this aim, a real-time qPCR-based approach was developed and applied to detect, quantify, and to type *L.* (*L*.) *infantum* as DEL and NonDEL in clinical material from different sources and hosts. The obtained results updated the distribution and relative frequency of the DEL and NonDEL genotypes along the Americas, offering an accurate and reliable qPCR-based approach able to quantify DEL and NonDEL parasites in clinical material, even in mixed infections.

## Methods

### Samples and DNA preparation

Samples were obtained based on availability, following a convenience sampling approach. Parasites were donated by the Coleção de *Leishmania* da FIOCRUZ (CLIOC—https://clioc.fiocruz.br). The *L.* (*L*.) *infantum* strains used as standards were previously characterized as DEL or NonDEL in a former study, either by qPCR or by whole-genome sequencing (WGS) [14, 17] (Additional file 1: Table S2). The strains were kept in culture and prepared for further DNA isolation by the High Pure PCR Template Preparation Kit (Roche) in accordance with the manufacturer protocol. The species was confirmed either by multilocus enzyme electrophoresis (MLEE), also in accordance with internal procedures or by PCR targeting ITS1 [21] followed by DNA sequencing. A total of 238 strains were used. The data include samples from different Brazilian states, Panama, and Honduras, from either human (*n* = 112) or dogs (*n* = 126). All additional details on hosts, voucher and international codes, geographic origin, the genotype, and availability as publicly available genomes are presented in Additional file 1: Table S2. The time span of the strain collection is1974–2017.

DNA from clinical samples was obtained from the Reference service and from studies and collaborators from Brazil and other American countries. The material was shipped to the Laboratório de Referência e Diagnóstico para *Leishmania* (LRDIL) to confirm the presence of *Leishmania* DNA and for the typing of the infecting species by molecular methods, i.e., conventional PCR targeting the ITS1 region of *Leishmania* sp. [21] followed by sequencing.

Samples include replicates from the same host or single samples. In total, 292 different individuals were included, and the panel was composed of samples from dogs (*n* = 369), cats (*n* = 2), coatis (*n* = 10), and phlebotomine sand flies (*n* = 3). The clinical material included fragments of healthy skin, skin lesions, bone marrow aspirate, lymph node aspirate and spleen, and, additionally, the intestines from the phlebotomine sand flies (Additional file 1: Table S3).

### qPCR and primers design

**Fig. 1:**
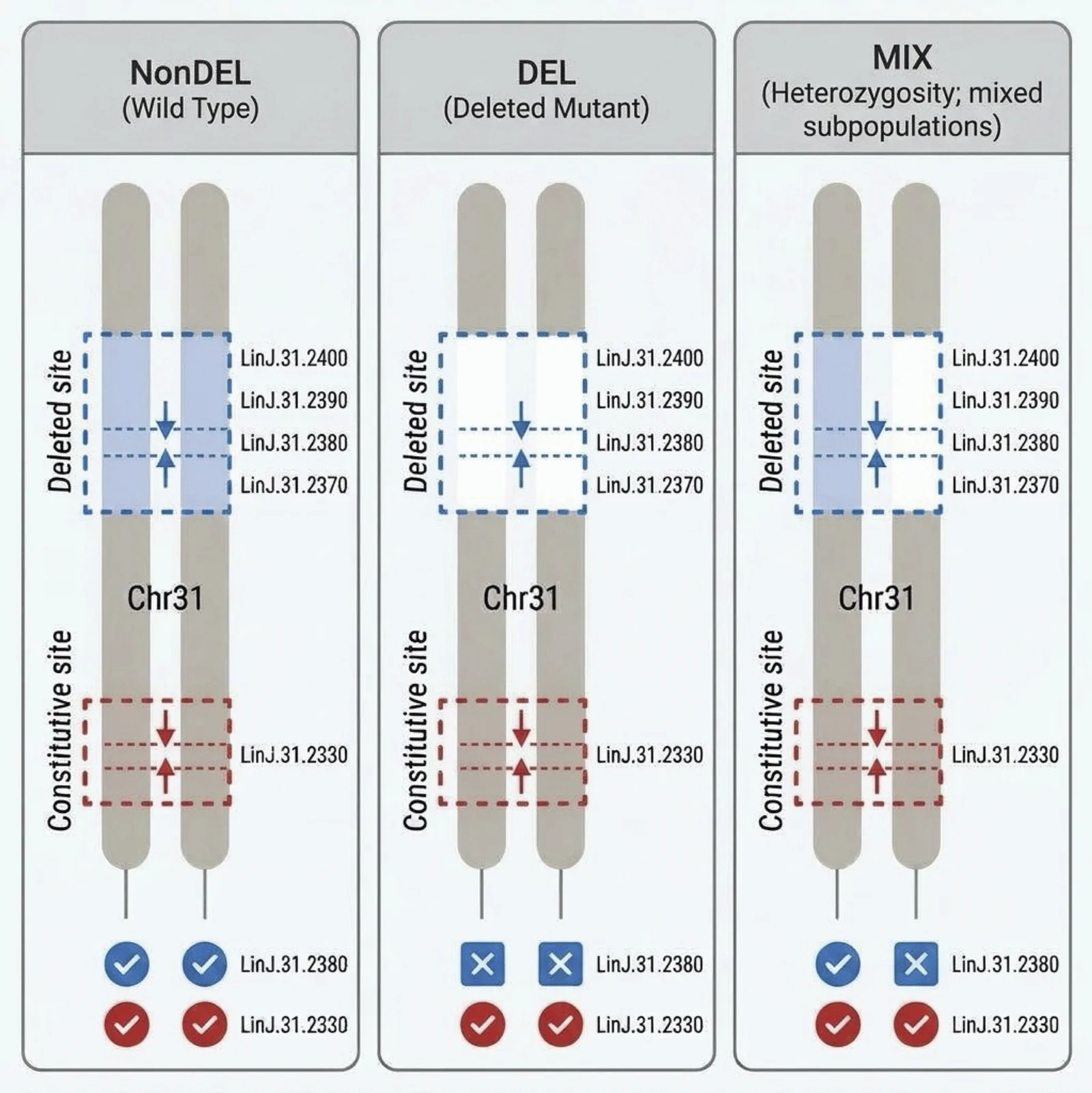
Schematic representation of chromosome 31 (gray bars) in *Leishmania* (*L*.) *infantum*, illustrating the NonDEL (wild-type), DEL (deletion mutant), and MIX (composed by heterozygous parasites or mixed subpopulations). The figure depicts the strategy used for primer design to differentially detect the genotypes. Primers targeting the constitutive region (LinJ.31.2330, shown in red) result in amplification in DEL, NonDEL, and “MIX” samples. In contrast, primers targeting the delete region (LinJ.31.2380, shown in blue) yield full amplification exclusively in NonDEL samples. Samples displaying a MIX qPCR profile show amplification pattern consistent with the presence of both targets, but Ct values may vary depending on the copy number of DEL and NonDEL Chromosomes. This profile may reflect heterozygosity, a mixed parasite population, or a combination of both, and does not allow discrimination between these possibilities. The figure was created using Gamma (https://gamma.app), an AI-assisted design platform

Primers and probes were designed targeting distinct regions of Chromosome 31, one of which is the 12 Kb deletion site described by our group and others [15]. The available nucleotide sequence for *L.* (*L*.) *infantum* JPCM5 genome-chromosome 31 was combined with other available genomes to allow the identification of conserved regions. To evaluate the deleted sub-chromosomic region, the NonDEL NCBI Reference Sequence NC_009415.2 (LINJ_31_2610 in TriTrypDB) was used as a template in Primer Blast (https://www.ncbi.nlm.nih.gov/tools/primer-blas). As a constitutive target, we selected a downstream region within chromosome 31 existent in both, DEL and NonDEL *L.* (*L.*) *infantum* strains. The chosen region (NCBI Reference Sequence: XM_001467491.1; NC_009415.2 in TriTrypDB) codes for metallo-peptidase, Clan MA(E), Family M1. Figure 1 depicts the strategy of using both targets to offer a typing diagnostic tool for infection by both *L.* (*L.*) *infantum* NonDEL and DEL genotypes and for complex samples that suggest DEL and NonDEL mixed parasite population, heterozygosity, or a combination of both. Primers and probes were named accordingly, i.e., 31_2330 for the constitutive site and 31_2380 for the deleted region.

The nucleotide sequences for primers and probes are depicted in Table 1 coded either as 31_2380—covering the deleted region, and 31_2330—the constitutive region. Available nucleotide sequences for the hypoxanthine phosphoribosyl-transferase 1 gene (HPRT) from domestic dog (*Canis lupus familiaris*) were used as template and as an endogenous target for the quantitative approach for clinical samples from dogs ( 1). For clinical material from other hosts, the 18S SSU target (conserved among mammals) was applied in conventional PCR and as an endogenous control to assess DNA quality and confirm the absence of PCR inhibition. Only samples in which the internal control amplified successfully were included in subsequent analyses.

**Table 1:** Primers and probes used to screen DEL and NonDEL genotypes in *Leishmania infantum*

Oligo ID	Nucleotide sequence (5'- 3')
^a^31_2380_Fw_Tq	5’-GCTGAAGTCAGTGAGCATGGA-3’
^a^31_2380_Rv_Tq	5’-TTCTGATCGTAGTGGTTGTGCA-3’
31_2330_Fw_Tq	5’-TCTGGTATGGAGCACCACTGC-3’
31_2330_Rv_Tq	5’-ATGAGCTCTGTCTGTTCCACCTC-3’
^a^31_2380_FAM_Tq	5'-FAM-ACTATTCTGCAGGGCCT-3'-NFQ-MGB
31_2330_VIC _Tq	5’-VIC-CGATTATTCTTAACGAGGCCA-3’-NFQ-MGB
HPRT Fw_canine	5'-GGA GGCCAT CACATCGTAGC-3'
HPRT Rv_canine	5'-AAT CCAGCA GGTCAGCAA AG-3'
HPRT_canine_FAM_Tq	5'-FAM-CAAGGGAGGCTATAAAT-NFQ/MGB-3'
^b^18s SSU Fw	5’ -CGGCTA CCA CATCCAAGG AA -3’
^b^18s SSU Rv	5’ -CCTGTATTGTTATTT TTC GTCACTACC T -3’

^a^Primers and probe targeting the deleted region

^b^Target conserved for all mammals applied in conventional PCR for clinical samples as an endogenous control to assess DNA quality and confirm the absence of PCR inhibition

### The qPCR protocol

The reaction volume of 20 µl includes the following: Mater Mix TaqMan system 1x (10 μL), 0.15 µM of each primer (0.3 μL), 0.100 µM of probes (0.2 μL), 7.2 μL DNAse/RNAse-free water, and 2 μL of DNA. The reaction conditions were as follows: Hot start of 95° for 10 min for 1 cycle; 45 cycles of 95° 15 s, 61^o^ 1 min. qPCRs were carried out with Applied Biosystems VIA7 equipment and Quant Studio Real-Time from the Plataforma de Análises Moleculares (RPT09J) (Rede de Plataformas Tecnológicas Fiocruz). A DNA-free master mix reaction (No Template Control—NTC) was included and all samples and controls were performed in duplicate.

### Genotyping setting

The genotyping option available in the software in the equipment Quant Studio 3 generates the Allelic Discrimination Plot, depicting the fluorescence detected by each probe (31_2330 and 31_2380) and clustering samples accordingly to the amplification efficiency and in accordance with selected standards. To apply this approach, the reactions described were multiplexed and the qPCR details are presented in Table 2.

**Table 2:** Reaction details for the multiplexed protocol prepared for the genotyping setting

Reagent	Concentration	Concentration/reaction	Unit	Volume/reaction µL
Water	–	–	–	6.4
Master Mix Taqman	2	1	X	10.0
31_2330_Fw_Tq	10	0.15	µM	0.3
31_2330_Rv_Tq	10	0.15	µM	0.3
31_2330_VIC_Tq	10	0.1	µM	0.2
31_2380_Fw_Tq	10	0.15	µM	0.3
31_2380_Rv_Tq	10	0.15	µM	0.3
31_2380_FAM_Tq	10	0.1	µM	0.2
DNA	–	–	–	2.0

### Mapping the distribution of genotypes

The mapping representation was obtained using the QGIS Geographic Information System, Open Source Geospatial Foundation Project [22]—http://qgis.osgeo.org). The input was prepared by the conversion of information of samples origins (locality) into geolocation data available for the municipality (Additional file: Tables S2 and S3). The frequencies of detection of DEL and NonDEL for each covered region were added in a table format used as in input for QGIS. The software then generates an output of the mapping of genotypes and the frequencies in a pie chart model.

### Determination of parasite load

Standard curves were obtained by the addition of 10^8^ parasites (strain IOC/L 2666; *L.* (*L*.) *infantum* NonDEL) into 20 mg of DNA derived from skin samples collected from non-infected dogs. The DNA of the material was extracted with the High Pure PCR Template Preparation Kit (Roche) in accordance with the manufacturer protocol. The DNA was diluted 1:10 in series of seven (starting from 10^8^ parasites into 20 mg) and included in the qPCR for each target (31_2330, 31_2380, and HPRT; and kDNA). The kDNA qPCR was applied to determine parasite load as a reference, a source of comparison to the new protocol tested due to its notorious high sensibility, derived from the great number of copies of the targeted region [23]. The primers used and protocol were published previously [23] and reproduced here.

## Results

### Genotypes were accurately detected by the protocol in various types of material

DNA from culture parasites representing a panel of *L.* (*L*.) *infantum* strains previously characterized as DEL and NonDEL by whole genome sequencing (WGS) [14] was used to validate the qPCR protocol as a tool to differentiate DEL and NonDEL genotypes (Table 3).

**Table 3:** Strains selected for protocol validation, including collection voucher (CLIOC), international code, genotype determined by WGS and confirmed by the present protocol, geographic origin, and host

Genotype	Voucher	International code	Geographic origin	Host
DEL	2769	MCAN/BR/2005/SPACK	PE	Canine
	2976	MHOM/BR/2006/1406MBS	PI	Human
	3116	MHOM/BR/2009/HU-UFS11	SE	Human
	3223	MCAN/BR/2010/BOLINHA-III	MT	Canine
	3227	MCAN/BR/2010/MAGRÃO	MT	Canine
	3598	MCAN/BR/2015/TUBO9	RJ	Canine
	3684	MHOM/BR/2006/ARS 3P	PI	Human
	3729	MCAN/BR/2016/PRETO	SC	Canine
	3634	MCAN/BR/2016/90	RJ	Canine
HTZ	3134	MCAN/BR/2009/GRANDÃO I	MT	Canine
	2949	MCAN/BR/2004/LIBPI-18	PI	Canine
	3254	MCAN/BR/2009/CLV17	SP	Canine
NonDEL	2666	MACAN/BR/2002/LVV-137	MS	Canine
	2848	MCAN/BR/2005/NMT-BSB159B	BA	Canine
	2972	MHOM/BR/2005/742EMS	PI	Human
	3124	MHOM/PT/1989/IMT-202	Portugal	Human
	3210	MCAN/BR/2010/CALSITO I	MT	Canine
	3556	MCAN/BR/2014/18MEDULA	MA	Canine
	3685	MHOM/BR/2006/MA03-A 4P(GM)	PI	Human
	2668	MCAN/BR/2002/LVV-139	MS	Canine
	2584	MHOM/BR/2003/JBIC	MS	Human
	2664	MCAN/BR/2002/LVV-135	MS	Canine
	3737	MCAN/BR/2017/BETHOVEN	SC	Canine

Parasites were obtained from CLIOC, and DNA was extracted from cultures using the QIAGEN kit. Species identification was confirmed by Multilocus Enzyme Electrophoresis (MLEE) and sequencing

*DEL* deleted, *NonDEL* not deleted, *HTZ* cloned parasites previously confirmed and heterozygotes by qPCR, *PE* Pernambuco, *PI* Piauí, *SE* Sergipe, *MT* Mato Grosso, *RJ* Rio de Janeiro, *SC* Santa Catarina, *SP* São Paulo, *MS* Mato Grosso do Sul, *BA* Bahia, *MA* Maranhão

**Fig. 2:**
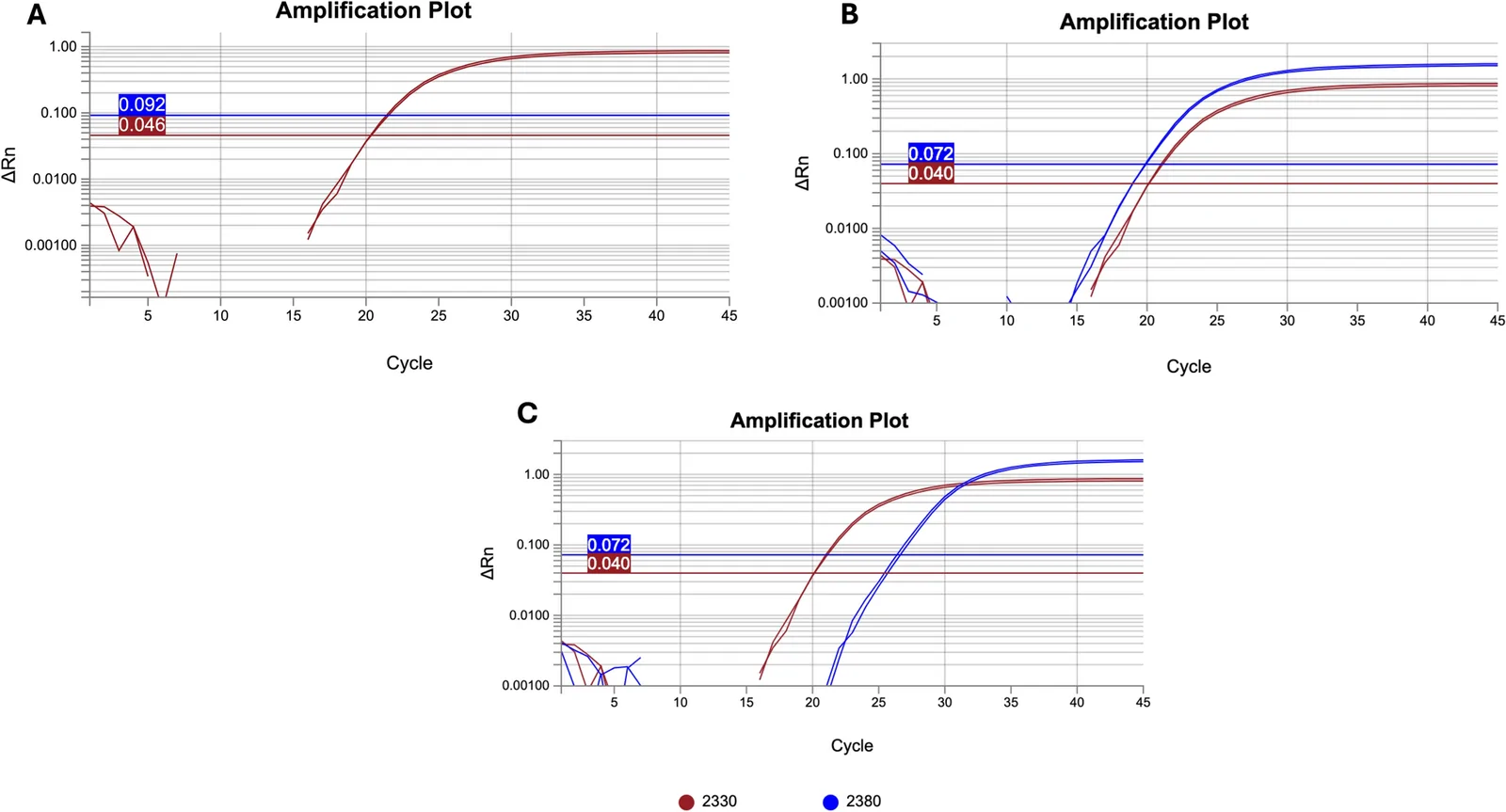
qPCR amplification plot of the constitutive target 31_2330 and the deleted site 31_2380 in reference samples for DEL, NonDEL, and HTZ genotypes. **A** DEL sample (IOCL3598) showing only the amplification of 2330 (constitutive site). **B** NonDEL sample (IOCL 2666) showing both amplifications with similar Cts values. **C** HTZ sample (IOCL 3134) showing the deviation between Cts (ΔCt) presents lesser number of copies due to the presence of the deletion only in half of the homologue chromosomes. The heterozygotes were only assumed for strain previously cloned and confirmed by quantitative qPCR. For clinical samples the term “MIX qPCR profile” was applied to describe samples with ΔCt values between those observed for DEL and NonDEL genotypes (**C**). This profile is consistent with either heterozygosity, mixed infection, or both, but does not allow discrimination between these possibilities

Results were interpreted after the confirmation of the amplification of the *Leishmania*-constitutive region 31_2330 in all samples (Fig. 2). Genotype was defined as DEL by the absence of 31_2380 amplification (Fig. 2A) and as NonDEL if 31_2380 also amplified (Fig. 2B). The outcome revealed 100% accuracy for detecting the genotypes (Table 3). Since amplification plots for both 31_2330 and 31_2380 show similar Ct values (Fig. 2B), samples exhibiting a Ct deviation (ΔCt > 0.5) between targets indicate non-equivalent target quantities. In previously cloned parasites, this pattern is consistent with heterozygosity, as shown in Fig. 2C. For clinical samples, however, ΔCt values falling between those observed for DEL and NonDEL genotypes are described as an “MIX qPCR profile.” This pattern is consistent with heterozygosity, mixed infection, or a combination of both, but does not allow discrimination between these possibilities.

### DEL parasites predominate and exhibit a broad geographic distribution across the Americas

After confirming the protocol as a typing tool for *L*. (*L*.) *infantum* genotypes a wider panel of samples (*n* = 622) was prepared including DNA from cultured parasites (*n* = 238) and clinical samples (n = 384) from different hosts and tissues. Most clinical samples in the present study are from dogs (*n* = 369), which are more accessible and a valuable material for surveillance. Therefore, specific primers and probes were also designed as internal constitutive targets for canine hosts’ DNA (HPRT), for use as sample-quality control and for parasite load (Table 4). For the clinical samples, the protocol presented a percentage of positivity of 90.2% compared to the conventional PCR based on ITS1 and allowed 72.9% of assayed clinical samples to be typed as DEL, NonDEL, and MIX/Htz.

**Table 4:** Number of samples assayed for the presented protocol and outcomes

	DEL	NonDEL	Mix/HTZ	Inconclusive	Negative	Total
Clinical samples	192 (50.0%)	69 (18.0%)	19 (4.9%)	68 (17.7%)	36 (9.4%)	384
Strains	170 (71.4%)	52 (21.8%)	16 (6.7%)	0	0	238
Total	362 (58.2%)	121 (19.5%)	35 (5.6%)	68 (10.9%)	38 (5.8%)	622

All clinical samples were previously confirmed by conventional PCR for the target ITS1 [21] or kDNA. DEL = 31_2330 amplified; 31_2380 negative. NonDEL = 31_2330 and 31_2380 amplified with almost overlapped Cts values (± 0.5). Mix/Htz = 31_2330 and 31_2380 amplified with Cts values deviating > 0.5. Inconclusive = refer to those samples among which amplification was detected for 31_2330 and/or 31_2380 with Cts > 35. Negative samples = no amplification detected

Among the typed strains, 112 of 238 were isolated from human patients (*Homo sapiens*) and 126 of 238 from dogs (*Canis familiaris*). Genotype frequencies were similar between hosts, with DEL strains accounting for 65% and 77% of isolates, respectively (Additional file1: Fig.S1).

**Fig. 3:**
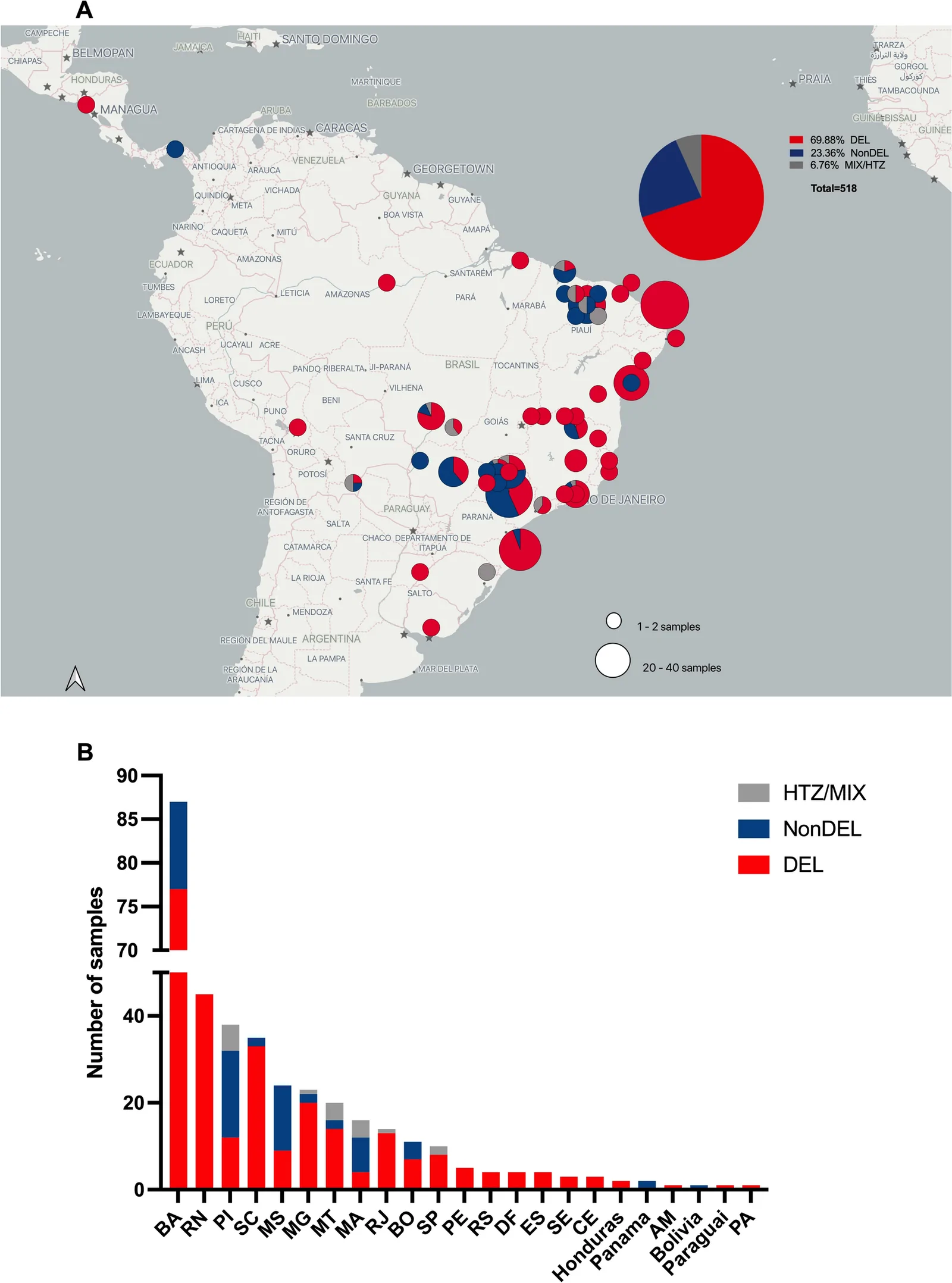
Map covering part of Central and South America showing the relative frequency and geographic distribution of genotypes and the absolute number of samples and genotypes per geographic origin. The panel of samples comprises both: strains (*n* = 238) from CLIOC and clinical material (*n* = 280). Inconclusive and negative samples were excluded and the remaining 518 samples (clinical and strains) mapped accordingly to the georeferenced locations from collecting data. The percentages reflect the higher frequency of DEL samples (depicted in the pie chart on the upper right). Figure with map and frequencies obtained by QGIS [22]. Basemap: Country and administrative boundaries derived from OpenStreetMap data (© OpenStreetMap contributors, Open Database License v1.0; https://www.openstreetmap.org), rendered using CARTO vector tiles (https://carto.com)

The distribution of the detected genotypes and their frequencies were mapped in Fig. 3A. The total of georeferenced localities (*n* = 57) included Brazil, Bolivia, Uruguay, Honduras, and Panama. The deleted genotype appeared more frequently (70%) and spread. Regions in Brazil, such as São Paulo, Mato Grosso, Mato Grosso do Sul, and Piaui, present more evidently the overlapping co-circulation of DEL and NonDEL. Moreover, samples from these regions commonly present a MIX qPCR profile (as depicted previously Fig. 2C), suggesting the co-infection by both genotypes, of presence of a heterozygous parasite population.

As the strains were obtained by convenience, the dataset carries the inherent biases associated with non-probabilistic sampling. The time span of the strain collection (1974–2017) is wide; however, the number of samples per year and their geographic origins vary greatly, limiting any meaningful temporal analysis.

### The genotyping qPCR setting differentially detected *L*. (*L*.) *donovani* complex species and identified DEL and NonDEL parasites in clinical samples

To test the accuracy of the Genotyping Method, a panel of DNAs from representative reference strains from different *Leishmania* species (representing *Leishmania* (*Viannia*) sp., *L.* (*Leishmania*) sp., *L*. (*Porcisia*), and Paraleishmania) was prepared for the multiplexed assay (Table 5). The software designs an Allelic Discrimination Plot, depicting the fluorescence detected by each probe (31_2330 and 31_2380) and clustering samples accordingly to the amplification efficiency. The clusters are color coded and the results are depicted in Table 5, in which the “Call” column depicts the software interpretation.

**Table 5:** Samples selected to test the differential genotyping approach by the real-time qPCR

Call	IOC/L	Species by MLEE	International code	Previous genotyping	Sample
No amplification	582	*L. (P) hertigi*	MCOE/PA/1965/C8	NA	Ref.
	1023	*L. (V) lainsoni*	MHOM/BR/1981/M6426	NA	Ref.
	888	*L. equatorensis*	MCHO/EC/1982/LSP1	NA	Ref.
	2821	*L. (L) major*	MHOM/IL/1980/FRIEDLIN	NA	Ref.
	581	*L. (L) major*	MHOM/SU/1973/5-ASKH	NA	Ref.
	561	*L. (L) mexicana*	MHOM/BZ/1982/BEL21	NA	Ref.
	571	*L. (L) tropica*	MHOM/SU/1958/STRAIN-OD	NA	Ref.
	566	*L. (V) braziliensis*	MHOM/BR/1975/M2903	NA	Ref.
	565	*L. (V) guyanensis*	MHOM/BR/1975/M4147	NA	Ref.
	1365	*L. (V) naiffi*	MDAS/BR/1979/M5533	NA	Ref.
	1545	*L. (V) shawi*	MCEB/BR/1984/M8408	NA	Ref.
	1245	*L. colombiensis*	IGOM/PA/1985/E582.34	NA	Ref.
Allele2/Allele2	3124	*L. (L) infantum*	MHOM/PT/1989/IMT-202	Ndel (Portugal)	Strain
	575	*L. (L) amazonensis*	IFLA/BR/1967/PH8	NA	Ref.
	563	*L. (L) donovani*	MHOM/ET/1967/HU3	NA	Ref.
	2848	*L. (L) infantum*	MCAN/BR/2005/NMT-BSB159B	Ndel (Bahia)	Strain
Allele1/Allele2	3210	*L. (L) infantum*	MCAN/BR/2010/CALSITO I	Ndel	Strain
	2666	*L. (L) infantum*	MACAN/BR/2002/LVV-137	Ndel	Strain
	2972	*L. (L) infantum*	MHOM/BR/2005/742EMS	Ndel	Strain
	2668	*L. (L) infantum*	MCAN/BR/2002/LVV-139	Ndel	Strain
	3685	*L. (L) infantum*	MHOM/BR/2006/MA03-A 4P(GM)	Ndel	Strain
	3134	*L. (L) infantum*	MCAN/BR/2009/GRANDÃO I	Htz	Strain
Allele1/Allele1	3598	*L. (L) infantum*	MCAN/BR/2015/TUBO9	Del	Strain
	3227	*L. (L) infantum*	MCAN/BR/2010/MAGRÃO	Del	Strain
	579	*L. (L) infantum*	MHOM/BR/1974/PP75	Del	Strain
	3116	*L. (L) infantum*	MHOM/BR/2009/HU-UFS11	Del	Strain
	2769	*L. (L) infantum*	MCAN/BR/2005/SPACK	Del	Strain

Representative samples from the DEL and NonDEL genotypes and reference strains from subgenus *L*. (*Viannia*), *L*. (*Leishmania*), *L*. (*Porcisia*), and Paraleishmania were included to cover a broad range of *Leishmania* sp. diversity. Call; IOC/L (voucher code); species determined by Multilocus Enzyme Electrophoresis (MLEE); International code; genotyping determined previously by WGS and/or qPCR; type of sample

**Fig. 4:**
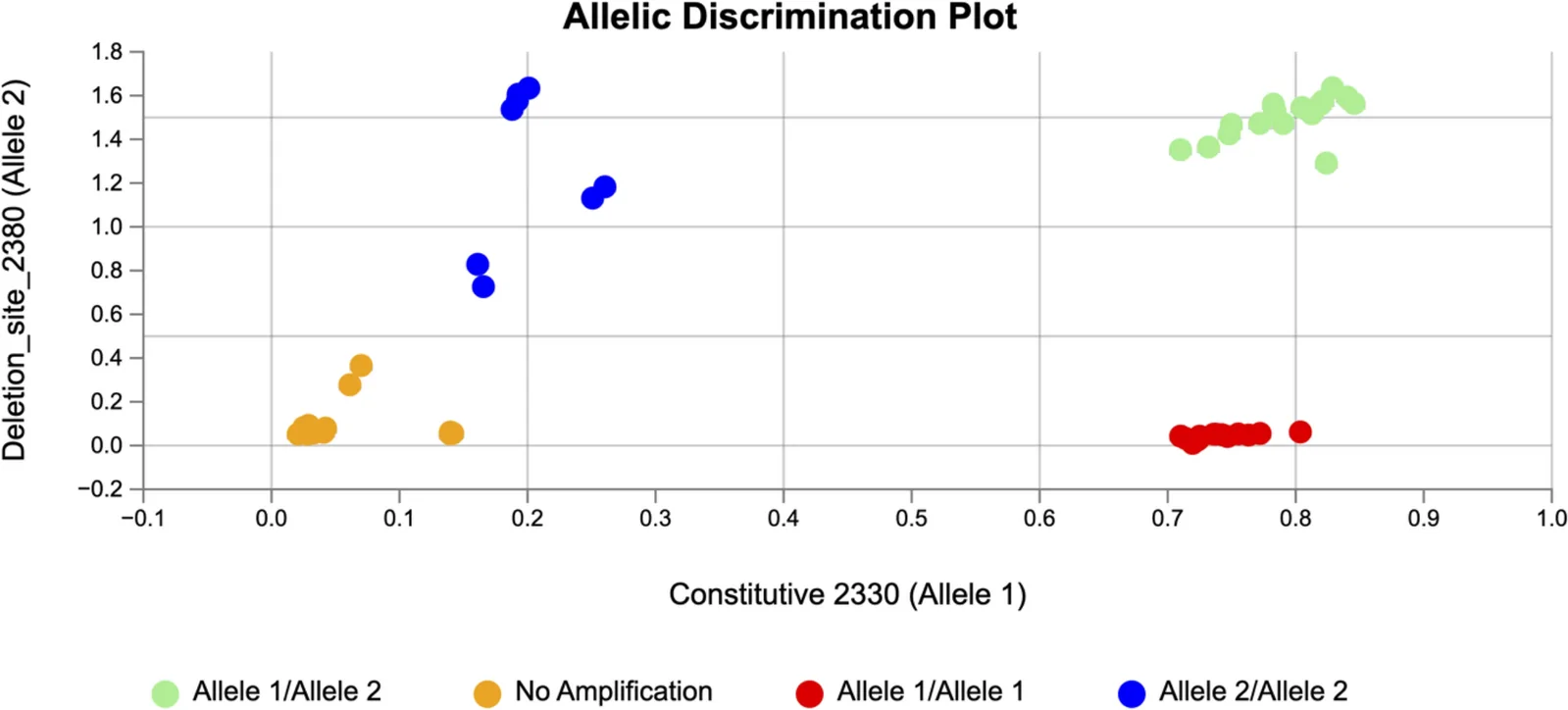
Allelic Discrimination Plot from the “Genotype” setting applied on DNA from culture parasite from the *L.* (*Viannia*), *L*. (*Porcisia*) Paraleishmania and *L*. (*Leishmania*) subgenus. Variable samples from the *L*. (*L*.) *donovani* complex were specially included to represent Old World and New World Del and NonDEL. In blue: *L*. (*L*.) *donovani*, *L*. (*L*.) *infantum* from the Old World (OW), and *L*. (*L*.) *amazonensis*; in green: the NonDEL and HTZ New World *L*. (*L*.) *infantum*; in red: the DEL samples from Brazil. The deleted site 31_2380 is fully amplified among blue and green samples, characterizing these strains as non-deleted; the conserved target 2330 is equally amplified for Brazilian DEL and NonDEL, confirming it as a good conserved endogenous target for genotyping New World samples. 31_2330 is not amplified among non-*L*. (*L*.) *donovani* complex samples, thus representing a potential marker for differential detection for these species; moreover, 2330 is only partially amplified among *L*. (*L*.) *donovani* and OW *L*. (*L*.). *infantum*, attending thus as a potential marker for geographic origin of the sample

The Plot (Fig. 4) depicted four color-coded groups: “ no amplification” (in orange), composed by all non-*L. donovani* complex species (except for *L.* (*L.*) *amazonensis*); “Allele2/Allele2” comprising the *L.* (*L*.) *donovani* reference strain, *L.* (*L*.) *amazonensis* and one *L.* (*L.*) *infantum* from the Old World (Portugal) and one from Bahia, Brazil—in blue; “Allele1/Allele2” formed by Brazilian *L.* (*L.*) *infantum* NonDEL and the HTZ sample (in green); and “Allele1/Allele1” formed by all Brazilian *L.* (*L*.) *infantum* DEL (in red). The deleted site 31_2380 was fully amplified among Old World and Brazilian NonDEL, confirming the genomic description for these samples as non-deleted. The 31_2330 target was fully amplified among all *L.* (*L.*) *infantum* Brazilian samples (DEL, NonDEL, and HTZ), but not fully amplified for the Old-World strain, supporting its use as an endogenous control marker for the reaction. This pattern may also allow differentiation between Old World and New World samples; however, we acknowledge that additional Old-World strains should be tested to confirm this observation. Importantly, although the 31_2330 locus is annotated in Old World reference genomes (*L.* (*L.*) *infantum* JPCM5, *L.* (*L*.) *donovani* BPK282A1, CL-SL, and HU3), the lack of amplification observed here may reflect sequence variation in primer annealing regions rather than true absence of the locus. Regardless of the underlying mechanism, the observed amplification profile remains informative for distinguishing among the tested samples. Notably, the 31_2330 target also allowed discrimination between members of the *L.* (*L*.) *donovani* complex and other *Leishmania* species assayed, except for *L.* (*L.*) *amazonensis*, which was also genotyped as “Allele2/Allele2.”

**Fig. 5:**
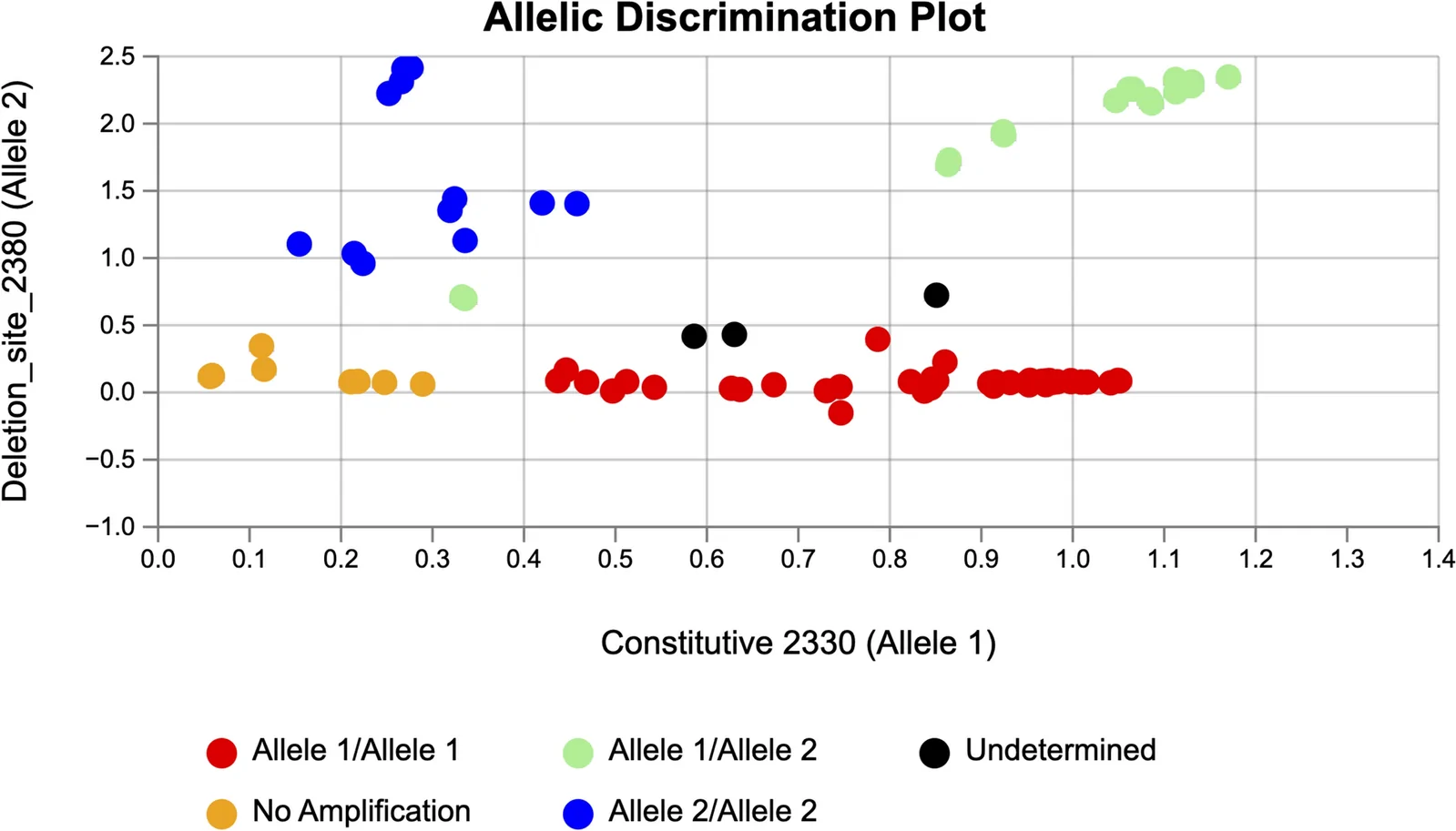
Allelic discrimination Plot for DEL, NonDEL reference samples (promastigotes) and clinical material. All clinical samples were previously confirmed as infected by *L*. (*L*.) *infantum* by conventional PCR for ITS1 target and sequencing. The DEL NonDEL genotype was first determined by qPCR applying absent/present amplification of targets 31.2380 and 31.2330

Additionally, to evaluate the performance of the assay in clinical material, 28 samples collected from 18 naturally infected dogs were analyzed (Table 6). To reflect the co-circulation of DEL and NonDEL parasites and the occurrence of mixed infections under field conditions, four samples previously classified as having a MIX qPCR profile were included. Genotypes had been previously determined by qPCR amplification of the 31_2380 target, as shown in Fig. 2. The Allelic Discrimination Plot (Fig. 5) confirmed the previous genotyping results for 21 of the 28 clinical samples (75.0%). The remaining seven samples (25.0%; Table 6, marked **) yielded inconclusive genotype assignments using the Genotyping Analysis setting. These samples had either been previously classified as having a MIX qPCR profile (4/7), consistent with the presence of both DEL and NonDEL genotypes or exhibited a low parasite load (Additional file 1: Fig. S3), which likely affected the software's ability to assign a genotype.

**Table 6:** Clinical samples selected to test the genotype method by the real-time qPCR

Animal	Genotyping call	Sample code	Species	Genotype	Tissue
1	Allele1/Allele1	17392 (1)	*L. (L) infantum*	Del	Skin
	Allele1/Allele1	17392 (2)	*L. (L) infantum*	Del	Skin–lesion
2	Allele1/Allele1	17393 (1)	*L. (L) infantum*	Del	Skin
	Allele1/Allele1	17393 (2)	*L. (L) infantum*	Del	Skin–lesion
3	Allele1/Allele1	18174 (1)	*L. (L) infantum*	Del	Skin
	Allele1/Allele2	18174 (2)	*L. (L) infantum*	Ndel	Skin–lesion
4	Allele1/Allele1	18445	*L. (L) infantum*	Del	Skin
5	Allele1/Allele1	18446	*L. (L) infantum*	Del	Skin
6	Allele1/Allele1	CHILI 8 PELE 1	*L. (L) infantum*	Del	Skin
	Allele1/Allele1	CHILI 8 PELE 2	*L. (L) infantum*	Del	Skin
7	Allele1/Allele1	^b^CALEJEIRO PELE 1	*L. (L) infantum*	Mix	Skin
8	Allele1/Allele1	^a^TRUENO PELE 1	*L. (L) infantum*	Mix	Skin
	Undetermined	^a^TRUENO PELE 1	*L. (L) infantum*	Mix	Skin
9	Allele1/Allele1	PELUSA 2 PELE 1	*L. (L) infantum*	Del	Skin
	Allele1/Allele1	PELUSA 2 PELE 2	*L. (L) infantum*	Del	Skin
	Allele1/Allele1	PELUSA 2 MO	*L. (L) infantum*	Del	Bone marrow
10	Allele1/Allele1	CHIQUILA PELE 1	*L. (L) infantum*	Del	Skin
	Allele1/Allele1	CHIQUILA MO	*L. (L) infantum*	Del	Bone marrow
11	Allele1/Allele2	18268	*L. (L) infantum*	Ndel	Skin
12	Allele1/Allele2	OSO PELE 1	*L. (L) infantum*	Ndel	Skin
	Allele1/Allele2	OSO PELE 2	*L. (L) infantum*	Ndel	Skin
13	No Amp	^b^18220	*L. (L) infantum*	Ndel	Skin
14	No Amp	^b^CACHO PELE 2	*L. (L) infantum*	Del	Skin
15	No Amp	^b^RUR 110 PELE 3T	*L. (L) infantum*	Del	Skin
16	Undetermined	^b^18225	*L. (L) infantum*	Mix	Skin
17	Allele2/Allele2	^c^CASO NOVO MO	*L. (L) infantum*	Ndel	Bone marrow
18	Allele2/Allele2	^c^PELUCHANA P2	*L. (L) infantum*	Ndel	Skin
	Allele2/Allele2	^c^PELUCHANA MO	*L. (L) infantum*	Ndel	Bone marrow

Twenty-eight samples from 18 animals were included. Sample code, species determined by conventional PCR and sequencing, the genotype previously determined, the Genotyping Call obtained by the software and tissue

^a^Same sample with different results between replicates

^b^Samples with divergent results between “Genotyping” and the beforehand qPCR-based typing result

^c^Samples confirmed as “NonDEL” but clustered in the *L. (L.) donovani* + OW group

### Parasite load is similar among animals infected with different *L. (L.) infantum* genotypes and those with a MIX qPCR profile

Standard curves were obtained for 31_2330 and 31_2380 *Leishmania* targets and for the canine HPRT gene included as the host tissue equivalent (Additional file 1: Fig. S1). The 28 samples from the Genotype setting (Table 6) and additional samples (total *n* = 20 DEL; *n* = 15 NonDEL; *n* = 5 MIX) were assayed using the 31_2330 protocol, and the results were compared with those from the available and highly sensitive protocol based on the kDNA target. The kDNA and 31_2330 presented a positive correlation (Pearson *r* = 0.98; Additional file 1: Table S1), but the mean value of equivalent of parasites/mg of tissue was higher by kDNA (1.689 for kDNA vs. 0.2414 for 31_2330), as expected due to the higher number of copies for this target (Additional file 1: Table S1). Bland–Altman test revealed poor agreement (Bias = −1.45, SD of Bias = 5.2) confirming that the 31_2330 protocol underestimates the parasite load.

**Fig. 6:**
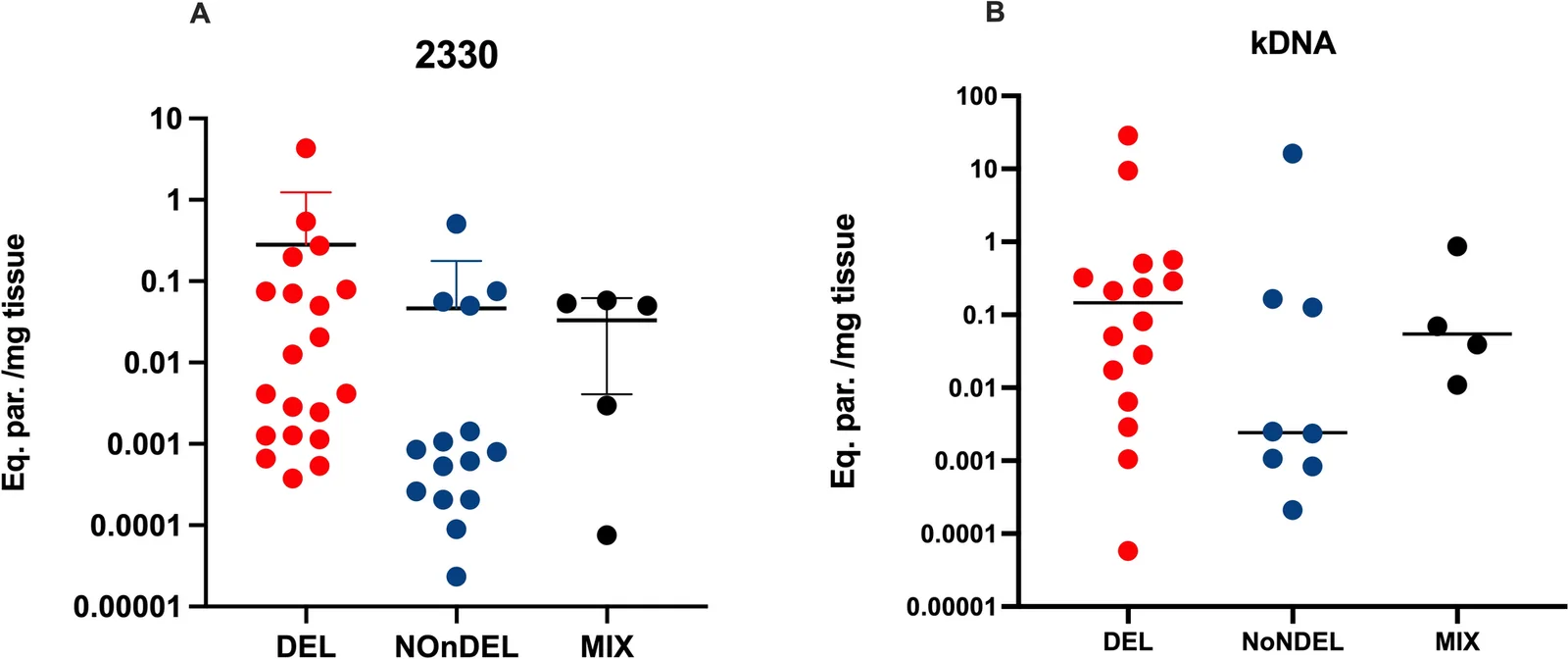
Parasite load in clinical samples infected with DEL, NonDEL, or mixed genotypes (MIX), quantified by **A** 31_2330 (*n* = 40) and **B** kDNA (*n* = 28) qPCR assays and normalized to HPRT. No significant differences were detected among groups (Kruskal–Wallis test, *P* = 0.053). Dots represent individual samples; lines indicate medians

Both protocols (31_2330 and kDNA) were applied to test the association between parasite load and the infecting genotype. No statistically significant difference in parasite load was detected among genotypes (Kruskal–Wallis, *P* = 0.053), although the observed trend suggests potential differences that may warrant further investigation with larger sample sizes (Fig. 6).

### Coinfections with both genotypes are observed

Paired samples from two dogs (18171 and 18174) and one cat, including lesion and healthy skin, were analyzed to compare genotype profiles within the same host (Table 7). For all animals, the healthy skin samples showed the DEL genotype, whereas the corresponding lesion samples displayed either the MIX qPCR profile or NonDEL. This pattern indicates differences in genotype profiles between anatomical sites within the same animal, suggesting underlying parasite heterogeneity.

**Table 7:** Paired samples from two dogs (18171 and 18174) and one cat (18520) showing distinct qPCR profiles between lesion and healthy skin

Animal	Sample	qPCR output	Municipality	State	Species
18520	Lesion (skin)	MIX qPCR profile	Rio de Janeiro	RJ	Cat
	Skin	DEL			
18174	Skin	DEL	Montes Claros	MG	Dog
	Lesion (skin)	NonDEL			
18171	Lesion (skin)	MIX qPCR profile	Montes Claros	MG	Dog
	Skin	DEL			

*RJ* Rio de Janeiro, *MG* Minas Gerais

## Discussion

The finding in the Americas of a mutant *L.* (*L.*) *infantum* carrying a 12 Kb deletion (DEL) in their genome co-circulating with NonDEL strains raise epidemiological questions on the effects of these parasites in the AVL context. To answer such queries a main step is to properly detect, map, and monitor the occurrence of DEL and NonDEL genotypes—the main goals of the present study.

We developed a qPCR-based strategy targeting (i) a “constitutive” genomic region, present and conserved among *L.* (*L.*) *infantum* strains, and (ii) the deleted region, which is amplified exclusively for the NonDEL, strain. The strategy proved to be accurate after being tested in DNA from cultured parasites previously characterized by WGS [14]. Then, aiming to obtain a mapping of the occurrence of genotypes, we selected a wider panel of strains deposited at CLIOC from distinct regions, including Central and South America countries. Our data confirm previous report of the broader and higher distribution of DEL parasites and their co-circulation with NonDEL parasites in several areas [14].

Importantly, we added to this panel DNA from clinical samples from various hosts and tissues, including those from Brazil and other South American countries. As presented before [14, 16], deletion-carrying *L.*(*L.*) *infantum* strains were more frequent and widely distributed, considering both the cultured parasites and the clinical material, thus excluding the potential in vitro-selection bias of testing only cultured parasites.

Clinical samples analyzed in this study were obtained primarily from dogs, with no human clinical samples included. This raises the question of whether the same genotype distribution is also present in human infections. To address this limitation, we examined strain-based data, which included isolates obtained from both humans and dogs, and found similar genotype frequencies across hosts. These findings suggest that the same *L. infantum* parasite population circulates in both humans and dogs, consistent with previous reports [1, 18, 24].

Although the relative contribution of dogs to sustaining *L. infantum* transmission remains debated, dogs are recognized as important hosts in the domestic transmission cycle, and associations between canine and human visceral leishmaniasis have been reported in several endemic settings [25, 26]. At the same time, transmission dynamics are influenced by multiple ecological and epidemiological factors, including vector biting rate, host feeding preferences, and host infectiousness, which have not been comprehensively evaluated [27, 28]. Consequently, the wide variation in canine visceral leishmaniasis seroprevalence reported across Brazil does not consistently parallel the incidence of human disease [25, 29]. Our findings therefore do not support preferential infection of dogs by the DEL genotype. Instead, they indicate that DEL and NonDEL parasites circulate in both host species. Nevertheless, because human clinical samples were not included in this study, it remains unclear whether the predominance of DEL parasites observed here is also reflected among human clinical infections, including asymptomatic or underdiagnosed cases. Future studies including human clinical samples will be important to address this question.

An additional implication of our findings concerns the use of miltefosine (MIL) for the treatment of canine visceral leishmaniasis. Although MIL was not approved for the treatment of human visceral leishmaniasis in Brazil because of its limited efficacy in clinical trials [15, 16], it has been authorized for the treatment of canine visceral leishmaniasis since 2016. Because DEL parasites have previously been associated with reduced susceptibility to MIL, the high frequency of this genotype observed in our study raises the question of whether parasite genotype may influence treatment outcomes in dogs. Although treatment efficacy has been evaluated both internationally [30] and in Brazil [19], none of these studies assessed the infecting parasite genotype. Future studies should therefore investigate whether treatment efficacy differs according to *L.* (*L.*) *infantum* genotype, an aspect that could have important implications for the clinical management of canine visceral leishmaniasis.

The sampling included a small number of biopsies from cats and coatis. Although these limited data do not allow epidemiological inferences, they underscore the need for further studies on these species and their potential roles in transmission cycles [31, 32], especially for the distinct genotypes.

Our sampling strategy presents some limitations. Because these are convenience samples, they do not necessarily represent all regions or transmission settings equally and therefore provide only relative genotype frequencies based on the material available. Moreover, it limits us to infer on factors such as regional variations in transmission dynamics, vector populations, reservoir hosts, or local treatment practices. In addition, the time span of sample collection was wide (1974–2017 for strains and 2022–2023 for clinical material). Although a temporal analysis would be informative, the number of samples per year and their geographic origins vary substantially, introducing considerable bias and limiting the reliability of such an approach. A more balanced dataset would be required for a meaningful temporal interpretation in future studies.

The MIX qPCR profile was observed among the clinical samples analyzed. In these cases, it is not possible to unequivocally distinguish between a mixed parasite population and a heterozygous genotype, particularly given the known polysomy of chromosome 31 in *L.* (*L.*) *infantum*, which can result in variable allele frequencies. Such MIX profiles are not unexpected, as heterozygous (HTZ) strains have been previously confirmed [14], and our mapping data indicate the co-circulation of DEL, NonDEL, and HTZ genotypes [14]. Analysis of paired samples from the same animal further supports this observation. In two dogs and one cat, healthy skin samples showed only the DEL genotype, whereas the corresponding lesion samples displayed either NonDEL or MIX qPCR profile. This pattern suggests variation in parasite composition across anatomical sites within the same host. Accordingly, the MIX qPCR profile is interpreted herein as indicative of underlying parasite heterogeneity, without allowing discrimination between the possibilities. The clinical and epidemiological implications of such heterogeneous infections remain unclear and represent an additional challenge for understanding disease dynamics.

Although not as sensitive, the equivalent of parasites per milligram of tissue obtained by the 31_2330 qPCR positively correlated with that obtained by the kDNA-based protocol. The approach, thus, allows to concomitantly identify and quantify the infecting genotype. This tool might be especially relevant to test associations between clinical outcomes vs. parasite load and infecting genotype. Having this in mind, we tested whether parasite load associates with genotypes. No difference was observed among groups, although the observed trend suggests potential differences that may warrant further investigation with larger sample sizes. Moreover, the assayed samples were from different localities, possibly from distinct transmission cycles. The same approach should thus be applied to numerically significant DEL and NonDEL samples from animals sharing the same region, preferably the same ecotype environment.

To offer the protocol as a typing tool and reduce subjectivity in genotype determination by operator evaluation—specifically through the observation of target 31_2380 amplification—and to facilitate the approach's adoption across different laboratories, we tested the "Genotype" option available in the equipment software. This option, which identifies SNPs within the region amplified by primer pairs 31_2330 and 31_2380, algorithmically clusters sample and assigns genotypes based on their plot positions. This methodology groups samples through a principal component analysis format based on multiplex target amplification. Representative strains from other species and subgenera were included to assess the protocol's potential as a differential diagnostic tool for *L.* (*L*.) *infantum* infection. The results showed high accuracy in distinguishing between DEL and NonDEL samples and potential to further differentiate *L.* (*L.*) *infantum* strains from the New World (Brazil) and Old World (Europe). Reference samples representing various *Leishmania* species clustered separately from the *L.* (*L.*) *donovani* complex, highlighting "Genotype" as an effective differential typing tool. An exception was *L.* (*L.*) *amazonensis*, part of the *L.* (*L.*) *mexicana* complex, which clustered with the *L.* (*L*.) *donovani* complex. The reasons for this distinction in *L.* (*L.*) *amazonensis* were not yet identified and represent a relevant drawback of the protocol, since reports of cutaneous leishmaniasis caused by *L.* (*L.*) *infantum* infection are available.

To test the “Genotype” approach on clinical material, we prioritized paired samples from the same animal, particularly those with distinct genotypes, as they provide the most reliable replicates to validate our approach. In practice, most duplicate samples from a single animal consisted of either two skin fragments or a combination of bone marrow and skin. The approach also showed good accuracy, though, as occurs in other methods, it could be affected by parasite load. Interestingly, two MIX samples were classified as "undetermined," with conflicting technical replicates, indicating the method's ability to detect complex samples containing mixtures.

## Conclusions

The qPCR-based molecular tool described here enables the simultaneous detection, discrimination, and quantification of the DEL, NonDEL, and MIX *Leishmania (L.) infantum* genotypes. Its application to a large and diverse collection of strains and clinical samples revealed the broad geographic distribution and predominance of the DEL genotype across the Americas, as well as the occurrence of mixed-genotype infections. Although consistent phenotypic differences among genotypes remain to be fully established, the DEL genotype has previously been associated with reduced susceptibility to miltefosine and other biological differences that warrant further investigation. These findings highlight the value of incorporating parasite genotyping into molecular surveillance studies and provide a framework for investigating associations between parasite genotype and clinically or epidemiologically relevant phenotypes. Given the use of miltefosine for the treatment of canine visceral leishmaniasis in Brazil, this approach may also contribute to future studies evaluating the impact of parasite genotype on therapeutic outcomes.

## Supplementary Information

Additional file 1: Fig. S1. Genotype distribution of DEL, NonDEL, and MIX* in human- and dog-derived strains visualized using complementary formats. Pie charts (A) illustrate the relative proportion of each genotype within each host, whereas bar plots (B) enable direct comparison across host groups. Statistical analysis revealed no significant association between host type and genotype frequency (chi-square test, p > 0.05). *MIX refers to samples with ΔCt values between those observed for DEL and NonDEL genotypes (MIX qPCR profile) or in-between read depth for whole genome sequencing data. The profile is consistent with either heterozygosity, mixed infection, or both, but does not allow discrimination between these possibilities. Fig. S2 Standard curves applied for parasite load. Targets 31_2330, HPRT, and 31_2380; Slop values and efficiency. Fig. S3. (A) Paired comparison of parasite loads, expressed as parasite equivalents per milligram of tissue, measured using the 2330 target and kDNA assays. Differences were assessed using the Wilcoxon matched-pairs signed-rank test. (B) Pearson correlation analysis showing a strong positive correlation between parasite loads obtained with the 2330 and kDNA targets (r = 0.98). Fig. S4. Distribution of parasite load according to concordance of genotyping results. Quartiles of parasite load were determined for samples with concordant and divergent results obtained using the genotyping approach. All samples with divergent results were restricted to the first (Q1) and second (Q2) quartiles, indicating that lower parasite loads are associated with reduced accuracy of the method. Table S1. Average values obtained for the parasite load determined by the 31_2330 and kDNA protocols. Table S2. Strains included in the study. Internal (Code 1) and International codes; voucher number from Coleção de Leishmania da FIOCRUZ (CLIOC); host; year of collection / isolation of parasites; Region / Brazilian state where the sample was collected; Municipality; georeferenced data (applied to obtain the Figure 3); genotype; how the genotype was obtained, either by Whole Genome Sequencing or by qPCR. Table S3. Metadata and genotyping results for all analyzed clinical samples. Columns include number of individuals (used to identify paired samples from the same host), indication of whether the sample is unique, sample ID, tissue type, geographical localization (I and II), host, and genotype result. Genotype classification is reported as DEL, NonDEL, inconclusive, or no amplification detected. In addition, a MIX qPCR profile was assigned to samples with ΔCt values intermediate between those observed for DEL and NonDEL genotypes, or with intermediate read depth in whole-genome sequencing data. This profile is consistent with heterozygosity, mixed infection, or both, but does not allow discrimination between these possibilities.

## Data Availability

All data supporting the findings of this study are available within this paper and its Supplementary Information.
