# Supplementary material for: Tracking the spread of a naturally occurring Leishmania (L.) infantum mutant: a qPCR-based investigation in strains and clinical samples
Source: Parasit Vectors. 2026 Aug 11;19:482. doi: 10.1186/s13071-026-07571-2 (PMC13746658; doi:10.1186/s13071-026-07571-2)
Supplement: Supplementary file 1 [file 13071_2026_7571_MOESM1_ESM.docx]

**Supplementary information**

**Additional file 1:**

**Fig. S1**. Genotype distribution of DEL, NonDEL, and MIX* in human- and dog-derived strains visualized using complementary formats. Pie charts (A) illustrate the relative proportion of each genotype within each host, whereas bar plots (B) enable direct comparison across host groups. Statistical analysis revealed no significant association between host type and genotype frequency (chi-square test, p > 0.05). *MIX refers to samples with ΔCt values between those observed for DEL and NonDEL genotypes (MIX qPCR profile) or in-between read depth for whole genome sequencing data. The profile is consistent with either heterozygosity, mixed infection, or both, but does not allow discrimination between these possibilities.

**Fig. S2**. Standard curves applied for parasite load. Targets 31_2330, HPRT and 31_2380; Slop values and efficiency.

**Fig. S3**.(A) Paired comparison of parasite loads, expressed as parasite equivalents per milligram of tissue, measured using the 2330 target and kDNA assays. Differences were assessed using the Wilcoxon matched-pairs signed-rank test. (B) Pearson correlation analysis showing a strong positive correlation between parasite loads obtained with the 2330 and kDNA targets (r = 0.98).

**Fig. S4.** Distribution of parasite load according to concordance of genotyping results. Quartiles of parasite load were determined for samples with concordant and divergent results obtained using the genotyping approach. All samples with divergent results were restricted to the first (Q1) and second (Q2) quartiles, indicating that lower parasite loads are associated with reduced accuracy of the method.

**Table S1.** Average values obtained for the parasite load determined by the 31_2330 and kDNA protocols.

|  | 2330 | kDNA |
| --- | --- | --- |
| Minimum | 8,93E-05 | 5,81E-05 |
| Median | 0.008352 | 0.0338 |
| Maximum | 4.306 | 28.7 |
| Mean | 0.2414 | 1.689 |
| Std. Deviation | 0.8742 | 6.065 |

| **Table S2.** Strains included in the study. Internal (Code 1) and International codes; voucher number from Coleção de Leishmania da FIOCRUZ (CLIOC); host; year of collection / isolation of parasites; Region / Brazilian state where the sample was collected; Municipality; georeferenced data (applied to obtain the Figure 3); genotype; how the genotype was obtained, either by Whole Genome Sequencing or by qPCR. | | | |  |  |  |  |  |  |  |
| --- | --- | --- | --- | --- | --- | --- | --- | --- | --- | --- |
| CODE 1 (internal) | Samples with voucher CLIOC | International code | HOST | Year of isolation | REGION / Brazilian state | Municipality | Latitu | Long | Genotype | Genome or qPCR |
| York | 3107 | MHOM/BR/2009/HU-UFS02 | Homo sapiens | 2009 | SE | Aracaju | -10,9167 | -37,0500 | DEL | GENOME |
| York | 3116 | MHOM/BR/2009/HU-UFS11 | Homo sapiens | 2009 | SE | Aracaju | -10,9167 | -37,0500 | DEL | GENOME |
| Del_SE_3595 | 3595 | MHOM/BR/2014/LVH5 | Homo sapiens | 2014 | SE | Aracaju | -10,9167 | -37,0500 | DEL | PCR |
| York | 3095 | MCAN/PY/2009/EG15 | Canis familiaris | 2015 | Paraguai | Asunción | -25,2485 | -57,5616 | DEL | GENOME |
| York | 3597 | MCAN/BR/2015/TUBO4 | Canis familiaris | 2015 | RJ | Barra Mansa | -22,5439 | -44,1708 | DEL | GENOME |
| Del_RJ_3598 | 3598 | MCAN_BR_2015_TUBO9 | Canis familiaris | 2015 | RJ | Barra Mansa | -22,5439 | -44,1708 | DEL | GENOME |
| Del_RJ_3634 | 3634 | MCAN_BR_2016_90 | Canis familiaris | 2016 | RJ | Barra Mansa | -22,5439 | -44,1708 | DEL | GENOME |
| Del_RJ_3635 | 3635 | MCAN_BR_2016_89 | Canis familiaris | 2016 | RJ | Barra Mansa | -22,5439 | -44,1708 | DEL | GENOME |
| York | 3635 | MCAN/BR/2016/89 | Canis familiaris | 2016 | RJ | Barra Mansa | -22,5439 | -44,1708 | DEL | GENOME |
| York | 2896 | MCER/BR/1979/M6445 | Canis familiaris | 1979 | PA | Belém | -1,4558 | -48,5039 | DEL | GENOME |
| York | 2906 | MHOM/BR/2002/LPC-RPV | Homo sapiens | 2002 | MG | Belo Horizonte | -19,9281 | -43,9419 | DEL | GENOME |
| Del_MG_3368 | 3368 | MHOM/BR/2011/AD1 | Homo sapiens | 2011 | MG | Belo Horizonte | -19,9281 | -43,9419 | DEL | PCR |
| Del_MG_3370 | 3370 | MHOM/BR/2011/CR1 | Homo sapiens | 2011 | MG | Belo Horizonte | -19,9281 | -43,9419 | DEL | PCR |
| York | 3374 | MHOM/BR/2012/AD4 | Homo sapiens | 2012 | MG | Belo Horizonte | -19,9281 | -43,9419 | DEL | GENOME |
| York | 3378 | MCAN/BR/2010/CA1 | Canis familiaris | 2010 | MG | Belo Horizonte | -19,9281 | -43,9419 | DEL | GENOME |
| York | 3379 | MCAN/BR/2010/CA2 | Canis familiaris | 2010 | MG | Belo Horizonte | -19,9281 | -43,9419 | DEL | GENOME |
| Del_MG_3380 | 3380 | MCAN_BR_2010_CA3 | Canis familiaris | 2010 | MG | Belo Horizonte | -19,9281 | -43,9419 | DEL | GENOME |
| York | 3380 | MCAN/BR/2010/CA3 | Canis familiaris | 2010 | MG | Belo Horizonte | -19,9281 | -43,9419 | DEL | GENOME |
| Del_MG_3381 | 3381 | MCAN_BR_2010_CA4 | Canis familiaris | 2010 | MG | Belo Horizonte | -19,9281 | -43,9419 | DEL | GENOME |
| York | 3381 | MCAN/BR/2010/CA4 | Canis familiaris | 2010 | MG | Belo Horizonte | -19,9281 | -43,9419 | DEL | GENOME |
| York | 2898 | MHOM/BR/2006/NMT-HUB402982MO | Homo sapiens | 2006 | DF | Brasília | -15,7939 | -47,8828 | DEL | GENOME |
| York | 2901 | MCAN/BR/2006/NMT-DF1421MO | Canis  familiaris | 2006 | DF | Brasília | -15,7939 | -47,8828 | DEL | GENOME |
| York | 2919 | MCAN/BR/2005/NMT-DF544MO | Canis familiaris | 2005 | DF | Brasília | -15,7939 | -47,8828 | DEL | GENOME |
| York | 3017 | MHOM/BR/2003/NMT-HUB 138030 | Homo sapiens | 2003 | DF | Brasília | -15,7939 | -47,8828 | DEL | GENOME |
| York | 2569 | MCAN/BR/2003/HUGER | Canis familiaris | 2003 | MS | Campo Grande | -20,4686 | -54,6222 | NonDEL | GENOME |
| York | 2584 | MHOM/BR/2003/JBIC | Homo sapiens | 2003 | MS | Campo Grande | -20,4686 | -54,6222 | NonDEL | GENOME |
| NonDel_MS_2664 | 2664 | MCAN_BR_2002_LVV-135 | Canis familiaris | 2002 | MS | Campo Grande | -20,4686 | -54,6222 | NonDEL | GENOME |
| York | 2665 | MCAN/BR/2002/LVV-136 | Canis familiaris | 2002 | MS | Campo Grande | -20,4686 | -54,6222 | NonDEL | GENOME |
| NonDel_MS_2688 | 2688 | MCAN_BR_2002_JACK_CUSTEAU | Canis familiaris | 2002 | MS | Campo Grande | -20,4686 | -54,6222 | NonDEL | GENOME |
| York | 2777 | MHOM/BR/2005/phufms-85 | Homo sapiens | 2005 | MS | Campo Grande | -20,4686 | -54,6222 | NonDEL | GENOME |
| York | 2778 | MHOM/BR/2005/phufms-89 | Homo sapiens | 2005 | MS | Campo Grande | -20,4686 | -54,6222 | NonDEL | GENOME |
| York | 2935 | MHOM/BR/2007/ARL | Homo sapiens | 2007 | MS | Campo Grande | -20,4686 | -54,6222 | NonDEL | GENOME |
| York | 3033 | MCAN/BR/2007/CG-1 | Canis familiaris | 2007 | MS | Campo Grande | -20,4686 | -54,6222 | DEL | GENOME |
| York | 3035 | MCAN/BR/2007/CG-2 | Canis familiaris | 2007 | MS | Campo Grande | -20,4686 | -54,6222 | NonDEL | GENOME |
| York | 329 | MCAN/BR/1984/C17.206 | Canis familiaris | 1984 | CE | Canindé, Ceará | -4,3589 | -39,3119 | DEL | GENOME |
| York | 603 | MCAN/BR/1984/CCC-17.481 | Canis familiaris | 1984 | CE | Caucaia | -3,7328 | -38,6558 | DEL | GENOME |
| York | 606 | MCAN/BR/1984/CCC17.482 | Canis familiaris | 1984 | CE | Caucaia | -3,7328 | -38,6558 | DEL | GENOME |
| York | 3132 | MCAN/BR/2009/PANTERA-MO-I | Canis familiaris | 2009 | MT | Cuiabá | -15,5958 | -56,0969 | DEL | GENOME |
| York | 3140 | MHOM/BR/2009/202 | Homo sapiens | 2009 | MT | Cuiabá | -15,5958 | -56,0969 | DEL | GENOME |
| York | 3158 | MCAN/BR/2009/113-MO | Canis familiaris | 2009 | MT | Cuiabá | -15,5958 | -56,0969 | DEL | GENOME |
| York | 3205 | MCAN/BR/2010/LEAO-I | Canis familiaris | 2010 | MT | Cuiabá | -15,5958 | -56,0969 | NonDEL | GENOME |
| York | 3208 | MCAN/BR/2010/ZEUS | Canis familiaris | 2010 | MT | Cuiabá | -15,5958 | -56,0969 | DEL | GENOME |
| York | 3209 | MCAN/BR/2010/ROBI | Canis familiaris | 2010 | MT | Cuiabá | -15,5958 | -56,0969 | DEL | GENOME |
| York | 3210 | MCAN/BR/2010/CALSITO-I | Canis familiaris | 2010 | MT | Cuiabá | -15,5958 | -56,0969 | NonDEL | GENOME |
| York | 3212 | MCAN/BR/2010/DIMY-I | Canis familiaris | 2010 | MT | Cuiabá | -15,5958 | -56,0969 | DEL | GENOME |
| Del_MT_3214 | 3214 | MCAN_BR_2010_MEG | Canis familiaris | 2010 | MT | Cuiabá | -15,5958 | -56,0969 | DEL | GENOME |
| York | 3219 | MCAN/BR/2010/TITÃ-I | Canis familiaris | 2010 | MT | Cuiabá | -15,5958 | -56,0969 | DEL | GENOME |
| York | 3223 | MCAN/BR/2010/BOLINHA-III | Canis familiaris | 2010 | MT | Cuiabá | -15,5958 | -56,0969 | DEL | GENOME |
| York | 3224 | MCAN/BR/2010/GUG | Canis familiaris | 2010 | MT | Cuiabá | -15,5958 | -56,0969 | HTZ** | GENOME |
| York | 3225 | MCAN/BR/2010/CHITARA-I | Canis familiaris | 2010 | MT | Cuiabá | -15,5958 | -56,0969 | DEL | GENOME |
| Del_MT_3227 | 3227 | MCAN_BR_2010_MAGRÃO | Canis familiaris | 2010 | MT | Cuiabá | -15,5958 | -56,0969 | DEL | GENOME |
| York | 3320 | MCAN/BR/2010/282-II | Canis familiaris | 2010 | MT | Cuiabá | -15,5958 | -56,0969 | DEL | GENOME |
| Del_SP_3250 | 3250 | MCAN_BR_2009_CLV9 | Canis familiaris | 2009 | SP | Embu das Artes | -23,6489 | -46,8519 | DEL | GENOME |
| HTZ_SP_3254 | 3254 | MCAN_BR_2009_CLV17 | Canis familiaris | 2009 | SP | Embu das Artes | -23,6489 | -46,8519 | in-between* | GENOME |
| York | 3254 | MCAN/BR/2009/CLV17 | Canis familiaris | 2009 | SP | Embu das Artes | -23,6489 | -46,8519 | HTZ** | GENOME |
| York | 3256 | MCAN/BR/2009/CLV22 | Canis familiaris | 2009 | SP | Embu das Artes | -23,6489 | -46,8519 | DEL | GENOME |
| York | 3257 | MCAN/BR/2011/IMTS-14 | Canis familiaris | 2011 | SP | Embu das Artes | -23,6489 | -46,8519 | DEL | GENOME |
| York | 3446 | MCAN/BR/2010/LSC_LOB | Canis familiaris | 2010 | SC | Florianópolis | -27,5933 | -48,5530 | DEL | GENOME |
| Del_SC_3716 | 3716 | MCAN/BR/2010/IRIS | Canis familiaris | 2010 | SC | Florianópolis | -27,5933 | -48,5530 | DEL | PCR |
| Del_SC_3717 | 3717 | MCAN/BR/2014/BOB | Canis familiaris | 2014 | SC | Florianópolis | -27,5933 | -48,5530 | DEL | PCR |
| Del_SC_3718 | 3718 | MCAN/BR/2015/SNOOPY | Canis familiaris | 2015 | SC | Florianópolis | -27,5933 | -48,5530 | DEL | PCR |
| York | 3719 | MCAN/BR/2015/PACO | Canis familiaris | 2015 | SC | Florianópolis | -27,5933 | -48,5530 | DEL | GENOME |
| Del_SC_3720 | 3720 | MCAN/BR/2014/FAISCA | Canis familiaris | 2014 | SC | Florianópolis | -27,5933 | -48,5530 | DEL | PCR |
| Del_SC_3721 | 3721 | MCAN/BR/2010/CHOCOLATE | Canis familiaris | 2010 | SC | Florianópolis | -27,5933 | -48,5530 | DEL | PCR |
| York | 3722 | MCAN/BR/2010/LOBA | Canis familiaris | 2010 | SC | Florianópolis | -27,5933 | -48,5530 | DEL | GENOME |
| Del_SC_3724 | 3724 | MCAN/BR/2014/NINNA | Canis familiaris | 2014 | SC | Florianópolis | -27,5933 | -48,5530 | DEL | PCR |
| Del_SC_3725 | 3725 | MCAN/BR/2015/TWISTY | Canis familiaris | 2015 | SC | Florianópolis | -27,5933 | -48,5530 | DEL | PCR |
| Del_SC_3726 | 3726 | MCAN/BR/2014/PINGO 2 | Canis familiaris | 2014 | SC | Florianópolis | -27,5933 | -48,5530 | DEL | PCR |
| Del_SC_3727 | 3727 | MCAN/BR/2015/KIARA 2 | Canis familiaris | 2015 | SC | Florianópolis | -27,5933 | -48,5530 | DEL | PCR |
| Del_SC_3728 | 3728 | MHOM/BR/2017/PC | Homo sapiens | 2017 | SC | Florianópolis | -27,5933 | -48,5530 | DEL | PCR |
| Del_SC_3729 | 3729 | MCAN/BR/2016/PRETO | Canis familiaris | 2016 | SC | Florianópolis | -27,5933 | -48,5530 | DEL | PCR |
| Del_SC_3730 | 3730 | MCAN/BR/2014/DORA | Canis familiaris | 2014 | SC | Florianópolis | -27,5933 | -48,5530 | DEL | PCR |
| Del_SC_3731 | 3731 | MCAN/BR/2010/ZEUS | Canis familiaris | 2010 | SC | Florianópolis | -27,5933 | -48,5530 | DEL | PCR |
| York | 3733 | MCAN/BR/2015/LAIKA | Canis familiaris | 2015 | SC | Florianópolis | -27,5933 | -48,5530 | DEL | GENOME |
| York | 3734 | MCAN/BR/2015/LUMA | Canis familiaris | 2015 | SC | Florianópolis | -27,5933 | -48,5530 | DEL | GENOME |
| Del_SC_3735 | 3735 | MCAN/BR/2010/PONGO | Canis familiaris | 2010 | SC | Florianópolis | -27,5933 | -48,5530 | DEL | PCR |
| York | 3735 | MCAN/BR/2010/PONGO | Canis familiaris | 2010 | SC | Florianópolis | -27,5933 | -48,5530 | DEL | GENOME |
| Del_SC_3736 | 3736 | MCAN/BR/2014/AMIGO | Canis familiaris | 2014 | SC | Florianópolis | -27,5933 | -48,5530 | DEL | PCR |
| York | 3737 | MCAN/BR/2017/BETHOVEN | Canis familiaris | 2017 | SC | Florianópolis | -27,5933 | -48,5530 | NonDEL | GENOME |
| York | 3738 | MCAN/BR/2016/NICK | Canis familiaris | 2016 | SC | Florianópolis | -27,5933 | -48,5530 | DEL | GENOME |
| York | 3739 | MCAN/BR/2015/BOMBOM | Canis familiaris | 2015 | SC | Florianópolis | -27,5933 | -48,5530 | DEL | GENOME |
| Del_SC_3740 | 3740 | MCAN/BR/2010/BAROLO | Canis familiaris | 2010 | SC | Florianópolis | -27,5933 | -48,5530 | DEL | PCR |
| York | 3741 | MCAN/BR/2015/SCOOBY | Canis familiaris | 2015 | SC | Florianópolis | -27,5933 | -48,5530 | DEL | GENOME |
| York | 3742 | MCAN/BR/2010/CÃO | Canis familiaris | 2010 | SC | Florianópolis | -27,5933 | -48,5530 | DEL | GENOME |
| York | 3743 | MCAN/BR/2010/CHANEL | Canis familiaris | 2010 | SC | Florianópolis | -27,5933 | -48,5530 | NonDEL | GENOME |
| York | 3744 | MCAN/BR/2015/BONECA | Canis familiaris | 2015 | SC | Florianópolis | -27,5933 | -48,5530 | DEL | GENOME |
| York | 3750 | MCAN/BR/2015/GORKIM | Canis familiaris | 2015 | SC | Florianópolis | -27,5933 | -48,5530 | DEL | GENOME |
| York | 3751 | MCAN/BR/2015/HANNA | Canis familiaris | 2015 | SC | Florianópolis | -27,5933 | -48,5530 | DEL | GENOME |
| York | 3752 | MCAN/BR/2017/JUJU | Canis familiaris | 2017 | SC | Florianópolis | -27,5933 | -48,5530 | DEL | GENOME |
| York | 3753 | MCAN/BR/2015/MAGRELO | Canis familiaris | 2015 | SC | Florianópolis | -27,5933 | -48,5530 | DEL | GENOME |
| York | 3754 | MCAN/BR/2016/BOLA | Canis familiaris | 2016 | SC | Florianópolis | -27,5933 | -48,5530 | DEL | GENOME |
| York | 3755 | MCAN/BR/2015/NICO | Canis familiaris | 2015 | SC | Florianópolis | -27,5933 | -48,5530 | DEL | GENOME |
| Del_BA_579 | 579 | MHOM/BR/1974/PP75 | Homo sapiens | 1974 | BA | Ituaçu | -13,8128 | -41,2969 | DEL | PCR |
| Del_AM_3118 | 3118 | MHOM/BR/2009/BLVD | Homo sapiens | 2009 | AM | Manaus | -3,1000 | -60,0167 | DEL | PCR |
| York | 3050 | MHOM/BR/2008/RN-04 | Homo sapiens | 2008 | RN | Natal, Rio Grande do Norte | -5,7950 | -35,2089 | DEL | GENOME |
| York | 3051 | MHOM/BR/2008/RN-05 | Homo sapiens | 2008 | RN | Natal, Rio Grande do Norte | -5,7950 | -35,2089 | DEL | GENOME |
| York | 3052 | MHOM/BR/2008/RN-03 | Homo sapiens | 2008 | RN | Natal, Rio Grande do Norte | -5,7950 | -35,2089 | DEL | GENOME |
| York | 3176 | MCAN/BR/2010/PV63 | Canis familiaris | 2010 | RN | Natal, Rio Grande do Norte | -5,7950 | -35,2089 | DEL | GENOME |
| York | 3177 | MCAN/BR/2010/PV64 | Canis familiaris | 2010 | RN | Natal, Rio Grande do Norte | -5,7950 | -35,2089 | DEL | GENOME |
| York | 3178 | MCAN/BR/2010/PV65 | Canis familiaris | 2010 | RN | Natal, Rio Grande do Norte | -5,7950 | -35,2089 | DEL | GENOME |
| York | 3182 | MCAN/BR/2010/PV69 | Canis familiaris | 2010 | RN | Natal, Rio Grande do Norte | -5,7950 | -35,2089 | DEL | GENOME |
| York | 3183 | MCAN/BR/2010/PV71 | Canis familiaris | 2010 | RN | Natal, Rio Grande do Norte | -5,7950 | -35,2089 | DEL | GENOME |
| York | 3184 | MCAN/BR/2010/PV72 | Canis familiaris | 2010 | RN | Natal, Rio Grande do Norte | -5,7950 | -35,2089 | DEL | GENOME |
| York | 3185 | MCAN/BR/2010/PV73 | Canis familiaris | 2010 | RN | Natal, Rio Grande do Norte | -5,7950 | -35,2089 | DEL | GENOME |
| York | 3186 | MCAN/BR/2010/PV74 | Canis familiaris | 2010 | RN | Natal, Rio Grande do Norte | -5,7950 | -35,2089 | DEL | GENOME |
| York | 3187 | MCAN/BR/2010/PV75 | Canis familiaris | 2010 | RN | Natal, Rio Grande do Norte | -5,7950 | -35,2089 | DEL | GENOME |
| York | 3264 | MCAN/BR/2010/PV82 | Canis familiaris | 2010 | RN | Natal, Rio Grande do Norte | -5,7950 | -35,2089 | DEL | GENOME |
| York | 3274 | MCAN/BR/2010/PV97 | Canis familiaris | 2010 | RN | Natal, Rio Grande do Norte | -5,7950 | -35,2089 | DEL | GENOME |
| York | 3275 | MCAN/BR/2010/PV98 | Canis familiaris | 2010 | RN | Natal, Rio Grande do Norte | -5,7950 | -35,2089 | DEL | GENOME |
| York | 3276 | MCAN/BR/2010/PV99 | Canis familiaris | 2010 | RN | Natal, Rio Grande do Norte | -5,7950 | -35,2089 | DEL | GENOME |
| Del_RN_3330 | 3330 | MHOM/BR/2011/Diag 1367 | Homo sapiens | 2011 | RN | Natal, Rio Grande do Norte | -5,7950 | -35,2089 | DEL | PCR |
| York | 3335 | MCAN/BR/2011/PV-128 | Canis familiaris | 2011 | RN | Natal, Rio Grande do Norte | -5,7950 | -35,2089 | DEL | GENOME |
| Del_RN_3336 | 3336 | MHOM/BR/2011/TC 03 | Homo sapiens | 2011 | RN | Natal, Rio Grande do Norte | -5,7950 | -35,2089 | DEL | PCR |
| Del_RN_3339 | 3339 | MHOM/BR/2011/TC 18 | Homo sapiens | 2011 | RN | Natal, Rio Grande do Norte | -5,7950 | -35,2089 | DEL | PCR |
| Del_RN_3340 | 3340 | MHOM/BR/2011/TC 28 | Homo sapiens | 2011 | RN | Natal, Rio Grande do Norte | -5,7950 | -35,2089 | DEL | PCR |
| Del_RN_3341 | 3341 | MHOM/BR/2011/TC 50 | Homo sapiens | 2011 | RN | Natal, Rio Grande do Norte | -5,7950 | -35,2089 | DEL | PCR |
| Del_RN_3342 | 3342 | MHOM/BR/2011/TC 65 | Homo sapiens | 2011 | RN | Natal, Rio Grande do Norte | -5,7950 | -35,2089 | DEL | PCR |
| Del_RN_3343 | 3343 | MHOM/BR/2011/TC 95 | Homo sapiens | 2011 | RN | Natal, Rio Grande do Norte | -5,7950 | -35,2089 | DEL | PCR |
| York | 3403 | MHOM/BR/2012/DIAG1502 | Homo sapiens | 2012 | RN | Natal, Rio Grande do Norte | -5,7950 | -35,2089 | DEL | GENOME |
| York | 3405 | MHOM/BR/2012/DIAG1521 | Homo sapiens | 2012 | RN | Natal, Rio Grande do Norte | -5,7950 | -35,2089 | DEL | GENOME |
| York | 3407 | MHOM/BR/2012/DIAG1551 | Homo sapiens | 2012 | RN | Natal, Rio Grande do Norte | -5,7950 | -35,2089 | DEL | GENOME |
| York | 3435 | MHOM/BR/2012/DIAG1586 | Homo sapiens | 2012 | RN | Natal, Rio Grande do Norte | -5,7950 | -35,2089 | DEL | GENOME |
| Del_HN_167 | outside CLIOC | MHOM_HN_1989_167 | Homo sapiens | 1989 | Honduras | Orocuina | 13,4833 | -87,1000 | DEL | GENOME |
| NonDel_PA_317 | outside CLIOC | MHOM_PA_1979_WR317 | Homo sapiens | 1979 | Panama | Panama City | 9,0000 | -79,5000 | NonDEL | GENOME |
| NonDel_PA_85 | outside CLIOC | MHOM_PA_1978_WR285 | Homo sapiens | 1978 | Panama | Panama City | 9,0000 | -79,5000 | NonDEL | GENOME |
| York | 3023 | MCAN/BR/2007/CP-14 | Canis familiaris | 2007 | ES | Pancas | -19,2250 | -40,8508 | DEL | GENOME |
| York | 3068 | MCAN/BR/2008/CP-18 | Canis familiaris | 2008 | ES | Pancas | -19,2250 | -40,8508 | DEL | GENOME |
| York | 3552 | MCAN/BR/2014/QUIKE | Canis familiaris | 2014 | SP | Santo Anastácio | -21,9769 | -51,6519 | DEL | GENOME |
| NonDel_BO_3756 | 3756 | MCAN/BO/2017/PQ102P | Canis familiaris | 2017 | Bolivia | Puerto Quijarro | -19,0039 | -57,7138 | NonDEL | PCR |
| York | 2647 | MHOM/BR/2003/ACS | Homo sapiens | 2003 | PE | Recife | -8,0539 | -34,8808 | DEL | GENOME |
| York | 2769 | MCAN/BR/2005/SPACK | Canis familiaris | 2005 | PE | Recife | -8,0539 | -34,8808 | DEL | GENOME |
| York | 2933 | MCAN/BR/2006/MAIKE | Canis familiaris | 2006 | PE | Recife | -8,0539 | -34,8808 | DEL | GENOME |
| York | 3053 | MHOM/BR/2008/RJS | Homo sapiens | 2008 | PE | Recife | -8,0539 | -34,8808 | DEL | GENOME |
| Del_PE_3434 | 3434 | MHOM/BR/2012/EJC | Homo sapiens | 2012 | PE | Recife | -8,0539 | -34,8808 | DEL | PCR |
| York | 45 | MCAN/BR/1980/CR3 | Canis familiaris | 1980 | RJ | Rio de Janeiro | -22,9111 | -43,2056 | DEL | GENOME |
| Del_RJ_3015 | 3015 | MHOM_BR_2007_WC | Homo sapiens | 2007 | RJ | Rio de Janeiro | -22,9111 | -43,2056 | DEL | GENOME |
| York | 3757 | MCAN/BR/2017/BETANIA | Canis familiaris | 2017 | RJ | Rio de Janeiro | -22,9111 | -43,2056 | DEL | GENOME |
| HTZ_MT_3134 | 3134 | MCAN_BR_2009_GRANDÃO I | Canis familiaris | 2009 | MT | Rondonópolis | -16,4708 | -54,6358 | in-between* | GENOME |
| York | 3135 | MCAN/BR/2009/GRANDÃO-II | Canis familiaris | 2009 | MT | Rondonópolis | -16,4708 | -54,6358 | HTZ** | GENOME |
| York | 3136 | MCAN/BR/2009/PATETA | Canis familiaris | 2009 | MT | Rondonópolis | -16,4708 | -54,6358 | DEL | GENOME |
| York | 3137 | MCAN/BR/2009/SOL | Canis familiaris | 2009 | MT | Rondonópolis | -16,4708 | -54,6358 | HTZ** | GENOME |
| York | 3138 | MCAN/BR/2009/BRONCRIS | Canis familiaris | 2009 | MT | Rondonópolis | -16,4708 | -54,6358 | DEL | GENOME |
| York | 2848 | MCAN/BR/2005/NMT-BSB159B | Canis familiaris | 2005 | BA | Salvador, Bahia | -12,9831 | -38,4928 | NonDEL | GENOME |
| Del_HN_336 | outside CLIOC | MHOM_HN_1993_336 | Homo sapiens | 1993 | Honduras | Puerto Rico |  |  | DEL | GENOME |
| York | 3536 | MCAN/BR/2014/CLARINHA | Canis familiaris | 2014 | SP | São José do Rio Preto | -20,8200 | -49,3789 | DEL | GENOME |
| York | 3537 | MCAN/BR/2014/CORAGEM | Canis familiaris | 2014 | SP | São José do Rio Preto | -20,8200 | -49,3789 | DEL | GENOME |
| York | 3092 | MHOM/BR/2009/LCS | Homo sapiens | 2009 | MA | São Luís, Maranhão | -2,5300 | -44,3028 | DEL | GENOME |
| York | 3105 | MHOM/BR/2009/ANC | Homo sapiens | 2009 | MA | São Luís, Maranhão | -2,5300 | -44,3028 | NonDEL | GENOME |
| York | 3504 | MCAN/BR/2013/001-MO | Canis familiaris | 2013 | MA | São Luís, Maranhão | -2,5300 | -44,3028 | HTZ** | GENOME |
| York | 3554 | MCAN/BR/2014/17BAÇO | Canis familiaris | 2014 | MA | São Luís, Maranhão | -2,5300 | -44,3028 | NonDEL | GENOME |
| York | 3555 | MCAN/BR/2014/18BAÇO | Canis familiaris | 2014 | MA | São Luís, Maranhão | -2,5300 | -44,3028 | NonDEL | GENOME |
| York | 3556 | MCAN/BR/2014/18MEDULA | Canis familiaris | 2014 | MA | São Luís, Maranhão | -2,5300 | -44,3028 | NonDEL | GENOME |
| York | 3557 | MCAN/BR/2014/19BAÇO | Canis familiaris | 2014 | MA | São Luís, Maranhão | -2,5300 | -44,3028 | HTZ** | GENOME |
| York | 3558 | MCAN/BR/2014/21BAÇO | Canis familiaris | 2014 | MA | São Luís, Maranhão | -2,5300 | -44,3028 | DEL | GENOME |
| York | 3559 | MCAN/BR/2014/POODLE-FÊMEA | Canis familiaris | 2014 | MA | São Luís, Maranhão | -2,5300 | -44,3028 | NonDEL | GENOME |
| York | 3560 | MCAN/BR/2014/20M | Canis familiaris | 2014 | MA | São Luís, Maranhão | -2,5300 | -44,3028 | NonDEL | GENOME |
| Del_MA_04A | outside CLIOC | MHOM_BR_06_MA04A | Homo sapiens | 2006 | MA | Codó | -4,4550 | -43,8858 | DEL | GENOME |
| Del_MA_07A | outside CLIOC | MHOM_BR_06_MA07A | Homo sapiens | 2006 | MA | Caxias, Maranhão | -4,8589 | -43,3558 | DEL | GENOME |
| MIX_MA_02A | outside CLIOC | MHOM_BR_05_MA02A | Homo sapiens | 2005 | MA | Codó | -4,4550 | -43,8858 | in-between* | GENOME |
| MIX_MA_05A | outside CLIOC | MHOM_BR_05_MA05A | Homo sapiens | 2005 | MA | Timon, Maranhão | -5,0939 | -42,8369 | in-between* | GENOME |
| NonDel_MA_01A | outside CLIOC | MHOM_BR_06_MA01A | Homo sapiens | 2006 | MA | Paraibano | -6,4328 | -43,9839 | NonDEL | GENOME |
| NonDel_MA_03A | outside CLIOC | MHOM_BR_06_MA03A | Homo sapiens | 2006 | MA | Timon, Maranhão | -5,0939 | -42,8369 | NonDEL | GENOME |
| York | 2949 | MCAN/BR/2004/LIBPI-18 | Canis familiaris | 2004 | PI | Teresina | -5,0949 | -42,8042 | HTZ | GENOME |
| NonDel_PI_2972 | 2972 | MHOM_BR_2005_742EMS | Homo sapiens | 2005 | PI | Teresina | -5,0949 | -42,8042 | NonDEL | GENOME |
| Del_PI_2976 | 2976 | MHOM_BR_2006_1406MBS | Homo sapiens | 2006 | PI | Teresina | -5,0949 | -42,8042 | DEL | GENOME |
| York | 3037 | MCAN/BR/2007/LIBPI-50 | Canis familiaris | 2007 | PI | Teresina | -5,0949 | -42,8042 | DEL | GENOME |
| NonDel_PI_3680 | 3680 | MHOM/BR/2006/6909 | Homo sapiens | 2006 | PI | Teresina | -5,0949 | -42,8042 | NonDEL | PCR |
| NonDel_PI_3681 | 3681 | MHOM/BR/2006/6912 | Homo sapiens | 2006 | PI | Teresina | -5,0949 | -42,8042 | NonDEL | PCR |
| NonDel_PI_3682 | 3682 | MHOM/BR/2006/P112A 4P NMV | Homo sapiens | 2006 | PI | Teresina | -5,0949 | -42,8042 | NonDEL | PCR |
| NonDel_PI_3683 | 3683 | MHOM/BR/2006/JSMG 3P | Homo sapiens | 2006 | PI | Teresina | -5,0949 | -42,8042 | NonDEL | PCR |
| Del_PI_3684 | 3684 | MHOM/BR/2006/ARS 3P | Homo sapiens | 2006 | PI | Teresina | -5,0949 | -42,8042 | DEL | PCR |
| NonDel_PI_3685 | 3685 | MHOM/BR/2006/MA03-A 4P (GMS) | Homo sapiens | 2006 | PI | Teresina | -5,0949 | -42,8042 | NonDEL | PCR |
| Del_PI_3684 | 3686 | MHOM/BR/2006/MA04A 4P JNN | Homo sapiens | 2006 | PI | Teresina | -5,0949 | -42,8042 | DEL | PCR |
| NonDel_PI_3687 | 3687 | MHOM/BR/2006/FFS 3P PI-05 | Homo sapiens | 2006 | PI | Teresina | -5,0949 | -42,8042 | NonDEL | PCR |
| NonDel_PI_3688 | 3688 | MHOM/BR/2006/P109A 4P JAS | Homo sapiens | 2006 | PI | Teresina | -5,0949 | -42,8042 | NonDEL | PCR |
| NonDel_PI_3692 | 3692 | MHOM/BR/2006/891 | Homo sapiens | 2006 | PI | Teresina | -5,0949 | -42,8042 | NonDEL | PCR |
| NonDel_PI_3693 | 3693 | MHOM/BR/2006/930 | Homo sapiens | 2006 | PI | Teresina | -5,0949 | -42,8042 | NonDEL | PCR |
| NonDel_PI_3694 | 3694 | MHOM/BR/2006/890 | Homo sapiens | 2006 | PI | Teresina | -5,0949 | -42,8042 | NonDEL | PCR |
| Del_PI_3695 | 3695 | MHOM/BR/2006/867 | Homo sapiens | 2006 | PI | Teresina | -5,0949 | -42,8042 | DEL | PCR |
| NonDel_PI_3696 | 3696 | MHOM/BR/2006/6889 | Homo sapiens | 2006 | PI | Teresina | -5,0949 | -42,8042 | NonDEL | PCR |
| NonDel_PI_3697 | 3697 | MHOM/BR/2006/6893 | Homo sapiens | 2006 | PI | Teresina | -5,0949 | -42,8042 | NonDEL | PCR |
| Del_PI_3698 | 3698 | MHOM/BR/2006/791 | Homo sapiens | 2006 | PI | Teresina | -5,0949 | -42,8042 | DEL | PCR |
| NonDel_PI_3699 | 3699 | MHOM/BR/2006/6905 | Homo sapiens | 2006 | PI | Teresina | -5,0949 | -42,8042 | NonDEL | PCR |
| NonDel_PI_3700 | 3700 | MHOM/BR/2006/6914 | Homo sapiens | 2006 | PI | Teresina | -5,0949 | -42,8042 | NonDEL | PCR |
| NonDel_PI_3702 | 3702 | MHOM/BR/2006/Pi 04A 4P DHRi | Homo sapiens | 2006 | PI | Teresina | -5,0949 | -42,8042 | NonDEL | PCR |
| Del_PI_3703 | 3703 | MHOM/BR/2006/AAS 3P | Homo sapiens | 2006 | PI | Teresina | -5,0949 | -42,8042 | DEL | PCR |
| NonDel_PI_3704 | 3704 | MHOM/BR/2006/AAS 4P | Homo sapiens | 2006 | PI | Teresina | -5,0949 | -42,8042 | NonDEL | PCR |
| Del_PI_3705 | 3705 | MHOM/BR/2006/Pi 11A 4P MBS | Homo sapiens | 2006 | PI | Teresina | -5,0949 | -42,8042 | DEL | PCR |
| NonDel_PI_3707 | 3707 | MHOM/BR/2006/MA01-A 4P | Homo sapiens | 2006 | PI | Teresina | -5,0949 | -42,8042 | NonDEL | PCR |
| MIX_PI_04A | outside CLIOC | MHOM/BR/05/PI04A | Homo sapiens | 2005 | PI | Valença do Piauí | -6,4078 | -41,7458 | in-between* | GENOME |
| MIX_PI_05A | outside CLIOC | MHOM/BR/06/PI05A | Homo sapiens | 2006 | PI | Valença do Piauí | -6,4078 | -41,7458 | in-between* | GENOME |
| MIX_PI_08A | outside CLIOC | MHOM/BR/05/PI08A | Homo sapiens | 2005 | PI | Altos, Piauí | -5,0389 | -42,4608 | in-between* | GENOME |
| MIX_PI_09A | outside CLIOC | MHOM/BR/05/PI09A | Homo sapiens | 2005 | PI | Nova Santa Rita | -29,8569 | -51,2739 | in-between* | GENOME |
| MIX_PI_10A | outside CLIOC | MHOM/BR/06/PI10A | Homo sapiens | 2006 | PI | Teresina | -5,0949 | -42,8042 | in-between* | GENOME |
| NonDel_PI_07A | outside CLIOC | MHOM/BR/06/PI07A | Homo sapiens | 2005 | PI | Piripiri, Piauí | -4,2728 | -41,7769 | NonDEL | GENOME |
| NonDel_PI_12A | outside CLIOC | MHOM/BR/05/PI12A | Homo sapiens | 2005 | PI | Lima Campos | -4,5200 | -44,4669 | NonDEL | GENOME |
| York | 2666 | MCAN/BR/2002/LVV-137 | Canis familiaris | 2002 | MS | Três Lagoas | -20,7511 | -51,6783 | NonDEL | GENOME |
| NonDel_MS_2668 | 2668 | MCAN_BR_2002_LVV-139 | Canis familiaris | 2002 | MS | Três Lagoas | -20,7511 | -51,6783 | NonDEL | GENOME |
| York | 2669 | MCAN/BR/2002/LVV-140 | Canis familiaris | 2002 | MS | Três Lagoas | -20,7511 | -51,6783 | NonDEL | GENOME |
| York | 2671 | MCAN/BR/2002/LVV-145 | Canis familiaris | 2002 | MS | Três Lagoas | -20,7511 | -51,6783 | NonDEL | GENOME |
| York | 2702 | MHOM/BR/2003/phufms-155 | Homo sapiens | 2003 | MS | Três Lagoas | -20,7511 | -51,6783 | NonDEL | GENOME |
| York | 3189 | MCAN/BR/2010/BURRINHO-I | Canis familiaris | 2010 | RS | Uruguaiana | -29,7550 | -57,0878 | DEL | GENOME |
| York | 3193 | MCAN/BR/2010/LEONA | Canis familiaris | 2010 | RS | Uruguaiana | -29,7550 | -57,0878 | DEL | GENOME |
| Del_RS_3196 | 3196 | MCAN_BR_2010_LUNA II | Canis familiaris | 2010 | RS | Uruguaiana | -29,7550 | -57,0878 | DEL | GENOME |
| York | 3198 | MCAN/BR/2010/LAIKA | Canis familiaris | 2010 | RS | Uruguaiana | -29,7550 | -57,0878 | DEL | GENOME |
| Del_ES_2788 | 2788 | MHOM_BR_2005_DRD | Homo sapiens | 2005 | ES | Vitória, Espírito Santo | -20,3106 | -40,2972 | DEL | GENOME |
| Del_ES_2789 | 2789 | MHOM_BR_2005_HRNS-1 | Homo sapiens | 2005 | ES | Vitória, Espírito Santo | -20,3106 | -40,2972 | DEL | GENOME |
| Del_MG_11A | outside CLIOC | MHOM_BR_05_MG11A | Homo sapiens | 2005 | MG | Montes Claros | -16,7322 | -43,8636 | DEL | GENOME |
| Del_MG_12A | outside CLIOC | MHOM_BR_05_MG12A | Homo sapiens | 2005 | MG | Montes Claros | -16,7322 | -43,8636 | DEL | GENOME |
| Del_MG_13A | outside CLIOC | MHOM_BR_05_MG13A | Homo sapiens | 2005 | MG | São Francisco, Minas Gerais | -15,9489 | -44,8639 | DEL | GENOME |
| Del_MG_15A | outside CLIOC | MHOM_BR_05_MG15A | Homo sapiens | 2005 | MG | Porteirinha | -15,7428 | -43,0278 | DEL | GENOME |
| Del_MG_16A | outside CLIOC | MHOM_BR_05_MG16A | Homo sapiens | 2005 | MG | São Francisco, Minas Gerais | -15,9489 | -44,8639 | DEL | GENOME |
| Del_MG_17A | outside CLIOC | MHOM_BR_05_MG17A | Homo sapiens | 2005 | MG | Montes Claros | -16,7322 | -43,8636 | DEL | GENOME |
| Del_MG_18A | outside CLIOC | MHOM_BR_05_MG18A | Homo sapiens | 2005 | MG | Montes Claros | -16,7322 | -43,8636 | DEL | GENOME |
| Del_MG_19A | outside CLIOC | MHOM_BR_05_MG19A | Homo sapiens | 2005 | MG | Catuji | -17,3119 | -41,5169 | DEL | GENOME |
| Del_PI_02A | outside CLIOC | MHOM/BR/06/PI02A | Homo sapiens | 2006 | PI | José de Freitas | -4,7558 | -42,5758 | DEL | GENOME |
| Del_PI_03A | outside CLIOC | MHOM/BR/06/PI03A | Homo sapiens | 2006 | PI | Cabeceiras | -15,7958 | -46,9269 | DEL | GENOME |
| Del_PI_01A | outside CLIOC | MHOM/BR/06/PI01A | Homo sapiens | 2006 | PI | José de Freitas | -4,7558 | -42,5758 | DEL | GENOME |
| Del_PI_11A | outside CLIOC | MHOM/BR/06/PI11A | Homo sapiens | 2006 | PI | José de Freitas | -4,7558 | -42,5758 | DEL | GENOME |
| Del_RN_12VLh | outside CLIOC | 12VLh | Homo sapiens | NA | RN | Natal, Rio Grande do Norte | -5,7950 | -35,2089 | DEL | GENOME |
| Del_RN_13VLh | outside CLIOC | 13VLh | Homo sapiens | NA | RN | Natal, Rio Grande do Norte | -5,7950 | -35,2089 | DEL | GENOME |
| Del_RN_14VLh | outside CLIOC | 14VLh | Homo sapiens | NA | RN | Natal, Rio Grande do Norte | -5,7950 | -35,2089 | DEL | GENOME |
| Del_RN_15VLd | outside CLIOC | 15VLd | Homo sapiens | NA | RN | Natal, Rio Grande do Norte | -5,7950 | -35,2089 | DEL | GENOME |
| Del_RN_16VLd | outside CLIOC | 16VLd | Homo sapiens | NA | RN | Natal, Rio Grande do Norte | -5,7950 | -35,2089 | DEL | GENOME |
| Del_RN_17VLd | outside CLIOC | 17VLd | Homo sapiens | NA | RN | Natal, Rio Grande do Norte | -5,7950 | -35,2089 | DEL | GENOME |
| Del_RN_18Ah | outside CLIOC | 18Ah | Homo sapiens | NA | RN | Natal, Rio Grande do Norte | -5,7950 | -35,2089 | DEL | GENOME |
| Del_RN_19VLh | outside CLIOC | 19VLh | Homo sapiens | NA | RN | Natal, Rio Grande do Norte | -5,7950 | -35,2089 | DEL | GENOME |
| Del_RN_1VLh90 | outside CLIOC | 1VLh90 | Homo sapiens | NA | RN | Natal, Rio Grande do Norte | -5,7950 | -35,2089 | DEL | GENOME |
| Del_RN_20VLh | outside CLIOC | 20VLh | Homo sapiens | NA | RN | Natal, Rio Grande do Norte | -5,7950 | -35,2089 | DEL | GENOME |
| Del_RN_2VLh90 | outside CLIOC | 2VLh90 | Homo sapiens | NA | RN | Natal, Rio Grande do Norte | -5,7950 | -35,2089 | DEL | GENOME |
| Del_RN_3VLh90 | outside CLIOC | 3VLh90 | Homo sapiens | NA | RN | Natal, Rio Grande do Norte | -5,7950 | -35,2089 | DEL | GENOME |
| Del_RN_4VLh90 | outside CLIOC | 4VLh90 | Homo sapiens | NA | RN | Natal, Rio Grande do Norte | -5,7950 | -35,2089 | DEL | GENOME |
| Del_RN_5VLh90 | outside CLIOC | 5VLh90 | Homo sapiens | NA | RN | Natal, Rio Grande do Norte | -5,7950 | -35,2089 | DEL | GENOME |
| Del_RN_6CLh | outside CLIOC | 6CLh | Canis familiaris | NA | RN | Natal, Rio Grande do Norte | -5,7950 | -35,2089 | DEL | GENOME |
| Del_RN_7VLd | outside CLIOC | 7VLd | Homo sapiens | NA | RN | Natal, Rio Grande do Norte | -5,7950 | -35,2089 | DEL | GENOME |
| Del_RN_9Ah | outside CLIOC | 9Ah | Homo sapiens | NA | RN | Natal, Rio Grande do Norte | -5,7950 | -35,2089 | DEL | GENOME |
| NonDel_MG_14A | outside CLIOC | MHOM_BR_05_MG14A | Homo sapiens | NA | MG | Montes Claros | -16,7322 | -43,8636 | NonDEL | GENOME |
| NonDel_MS_MAM | outside CLIOC | MHOM_BR_2003_MAM | Homo sapiens | 2003 | MS | Campo Grande | -20,4686 | -54,6222 | NonDEL | GENOME |

(*) “In-between” refers to isolates displaying an intermediate genomic profile, characterized by approximately 50% read coverage across the deleted region relative to flanking regions, which may reflect either heterozygosity or a mixed population;

(**) “Heterozygous (HTZ)” is used exclusively for strains that were previously cloned and in which heterozygosity was experimentally confirmed by quantitative qPCR targeting the deleted region;

| **Table S3.** Metadata and genotyping results for all analyzed clinical samples. Columns include: number of individuals (used to identify paired samples from the same host), indication of whether the sample is unique, sample ID, tissue type, geographical localization (I and II), host, and genotype result. Genotype classification is reported as DEL, NONDEL, inconclusive, or no amplification detected. In addition, a MIX qPCR profile was assigned to samples with ΔCt values intermediate between those observed for DEL and NONDEL genotypes, or with intermediate read depth in whole-genome sequencing data. This profile is consistent with heterozygosity, mixed infection, or both, but does not allow discrimination between these possibilities. | | | |  |  |  |  |
| --- | --- | --- | --- | --- | --- | --- | --- |
| Number of individuals | Unique sample? | Sample ID | Tissue | Localization I | Localization II | Host | Genotype result |
| 1 | Yes | 17831 | Skin | Resende, Rio de Janeiro | Resende | Canine | DEL |
| 2 | Yes | 17893 | Skin | Rio de Janeiro | Rio de Janeiro | Canine | DEL |
| 3 | Yes | 17899 | Skin | Rio de Janeiro | Rio de Janeiro | Canine | DEL |
| 4 | No | 17392 (1) | Skin | Rio de Janeiro | Rio de Janeiro | Canine | DEL |
| 4 | No | 17392 (2) | Skin | Rio de Janeiro | Rio de Janeiro | Canine | DEL |
| 5 | No | 17393 (1) | Skin | Rio de Janeiro | Rio de Janeiro | Canine | DEL |
| 5 | No | 17393 (2) | Skin | Rio de Janeiro | Rio de Janeiro | Canine | DEL |
| 6 | Yes | 17905 | Skin | Belford Roxo, Rio de Janeiro | Belford Roxo | Canine | DEL |
| 7 | Yes | 17906 | Skin | Belford Roxo, Rio de Janeiro | Belford Roxo | Canine | DEL |
| 8 | No | 17902 (1) | Skin | Itatiaia | Itatiaia | Canine | DEL |
| 8 | No | 17902 (2) | Limph node | Itatiaia | Itatiaia | Canine | DEL |
| 9 | Yes | 17973 | Skin | Rio de Janeiro | Rio de Janeiro | Canine | DEL |
| 10 | Yes | 18084 | Skin | Rio de Janeiro | Santa Tereza - RJ | Canine | DEL |
| 11 | Yes | 18113 | Skin | Rio de Janeiro | Quintino Bocaiuva - RJ | Canine | DEL |
| 12 | Yes | 18124 | Skin | Rio de Janeiro | Engenho Novo - RJ | Canine | DEL |
| 13 | Yes | 18125 - BOB | Skin | Rio de Janeiro | Rio de Janeiro - RJ | Canine | DEL |
| 14 | Yes | 18169/LBT 13210 | Lesion (skin) | Montes Claros | Montes Claros - MG | Canine | NonDEL |
| 15 | Yes | 18170/LBT 13213 | Lesion (skin) | Montes Claros | Montes Claros - MG | Canine | DEL |
| 16 | No | 18171 (1)/LBT 13214 | Lesion (skin) | Montes Claros | Montes Claros - MG | Canine | MIX qPCR profile |
| 16 | No | 18171 (2)/LBT 13214 | Skin | Montes Claros | Montes Claros - MG | Canine | DEL |
| 17 | Yes | 18172/LBT 13215 | Skin | Montes Claros | Montes Claros - MG | Canine | MIX qPCR profile |
| 18 | No | 18174 (1)/LBT 13217 | Skin | Montes Claros | Montes Claros - MG | Canine | DEL |
| 18 | No | 18174(2)/LBT 13217 | Lesion (skin) | Montes Claros | Montes Claros - MG | Canine | NonDEL |
| 19 | Yes | 18175/LBT 13218 | Lesion (skin) | Montes Claros | Montes Claros - MG | Canine | NonDEL |
| 20 | Yes | 18176/LBT 13225 | Lesion (skin) | Montes Claros | Montes Claros - MG | Canine | NonDEL |
| 21 | Yes | 18177/LBT 13232 | Lesion (skin) | Montes Claros | Montes Claros - MG | Canine | NonDEL |
| 22 | Yes | 18220 | Skin | Rio de Janeiro | Rio de Janeiro | Canine | NonDEL |
| 23 | Yes | 18225 | Skin | Rio de Janeiro | Rio de Janeiro | Canine | MIX qPCR profile |
| 24 | Yes | 18243 | Skin | Rio de Janeiro | Rio de Janeiro | Canine | Inconclusive |
| 25 | Yes | 18268 | Skin | Rio de Janeiro | Rio de Janeiro | Canine | NonDEL |
| 26 | Yes | 18445 | Skin | Rio de Janeiro | Rio de Janeiro | Canine | DEL |
| 27 | Yes | 18446 | Skin | Rio de Janeiro | Rio de Janeiro | Canine | DEL |
| 28 | No | 18520 (1) | Lesion (skin) | Rio de Janeiro | Rio de Janeiro | Cat | MIX qPCR profile |
| 28 | No | 18520 (2) | Skin | Rio de Janeiro | Rio de Janeiro | Cat | DEL |
| 29 | Yes | CACHO PELE 2 | Skin | Villamontes | Villamontes -Tarija - Bolívia | Canine | DEL |
| 30 | Yes | CASO NOVO MO | Bone marrow | Villamontes | Villamontes -Tarija - Bolívia | Canine | NonDEL |
| 31 | No | CHILI 8 PELE 1 | Skin | Villamontes | Villamontes -Tarija - Bolívia | Canine | DEL |
| 31 | No | CHILI 8 PELE 2 | Skin | Villamontes | Villamontes -Tarija - Bolívia | Canine | DEL |
| 32 | Yes | CALEJEIRO PELE 1 | Skin | Villamontes | Villamontes -Tarija - Bolívia | Canine | MIX qPCR profile |
| 33 | Yes | TRUENO PELE 1 | Skin | Villamontes | Villamontes -Tarija - Bolívia | Canine | MIX qPCR profile |
| 34 | No | PELUSA 2 PELE 1 | Skin | Villamontes | Villamontes -Tarija - Bolívia | Canine | DEL |
| 34 | No | PELUSA 2 PELE 2 | Skin | Villamontes | Villamontes -Tarija - Bolívia | Canine | DEL |
| 34 | No | PELUSA 2 MO | Bone marrow | Villamontes | Villamontes -Tarija - Bolívia | Canine | DEL |
| 35 | No | PELUCHNA PELE 2 | Skin | Villamontes | Villamontes -Tarija - Bolívia | Canine | NonDEL |
| 35 | No | PELUCHNA MO | Bone marrow | Villamontes | Villamontes -Tarija - Bolívia | Canine | NonDEL |
| 36 | No | OSO PELE 1 | Skin | Villamontes | Villamontes -Tarija - Bolívia | Canine | NonDEL |
| 36 | No | OSO PELE 2 | Skin | Villamontes | Villamontes -Tarija - Bolívia | Canine | NonDEL |
| 37 | No | CHIQUILA PELE 1 | Skin | Villamontes | Villamontes -Tarija - Bolívia | Canine | DEL |
| 37 | No | CHIQUILA MO | Bone marrow | Villamontes | Villamontes -Tarija - Bolívia | Canine | DEL |
| 38 | No | RUR 49 PELE 2T | Skin | Camaçari | Camaçari - Bahia | Canine | DEL |
| 38 | No | RUR 49 BACO 1T | Spleen | Camaçari | Camaçari - Bahia | Canine | DEL |
| 38 | No | RUR 49 BACO 2T | Spleen | Camaçari | Camaçari - Bahia | Canine | DEL |
| 38 | No | RUR 49 PELE | Skin | Camaçari | Camaçari - Bahia | Canine | Inconclusive |
| 39 | Yes | RUR 140 PELE | Skin | Camaçari | Camaçari - Bahia | Canine | DEL |
| 40 | Yes | RUR 143 PELE | Skin | Camaçari | Camaçari - Bahia | Canine | No amplification detected |
| 41 | Yes | RUR 147 PELE | Skin | Camaçari | Camaçari - Bahia | Canine | No amplification detected |
| 42 | Yes | RUR 148 PELE | Skin | Camaçari | Camaçari - Bahia | Canine | DEL |
| 43 | Yes | RUR 234 PELE 2T | Skin | Camaçari | Camaçari - Bahia | Canine | No amplification detected |
| 44 | Yes | RUR 109 PELE | Skin | Camaçari | Camaçari - Bahia | Canine | Inconclusive |
| 45 | No | RUR 291 BACO | Spleen | Camaçari | Camaçari - Bahia | Canine | DEL |
| 45 | No | RUR 291 PELE | Skin | Camaçari | Camaçari - Bahia | Canine | DEL |
| 45 | No | RUR 291 BACO 2T | Spleen | Camaçari | Camaçari - Bahia | Canine | DEL |
| 46 | No | RUR 301 PELE | Skin | Camaçari | Camaçari - Bahia | Canine | No amplification detected |
| 46 | No | RUR 301 PELE 2T | Skin | Camaçari | Camaçari - Bahia | Canine | DEL |
| 47 | No | RUR 324 BACO 2T | Spleen | Camaçari | Camaçari - Bahia | Canine | No amplification detected |
| 47 | No | RUR 324 BACO 4T | Spleen | Camaçari | Camaçari - Bahia | Canine | DEL |
| 47 | No | RUR 324 BACO 3T | Spleen | Camaçari | Camaçari - Bahia | Canine | DEL |
| 47 | No | RUR 324 BACO | Spleen | Camaçari | Camaçari - Bahia | Canine | DEL |
| 48 | Yes | RUR 351 PELE 3T | Skin | Camaçari | Camaçari - Bahia | Canine | DEL |
| 49 | No | RUR 490 BACO | Spleen | Camaçari | Camaçari - Bahia | Canine | No amplification detected |
| 49 | No | RUR 490 BACO 1T | Spleen | Camaçari | Camaçari - Bahia | Canine | DEL |
| 50 | No | RUR 433 BACO 4T | Spleen | Camaçari | Camaçari - Bahia | Canine | DEL |
| 50 | No | RUR 433 BACO 3T | Spleen | Camaçari | Camaçari - Bahia | Canine | DEL |
| 51 | No | RUR 65 PELE 2T | Skin | Camaçari | Camaçari - Bahia | Canine | DEL |
| 51 | No | RUR 65 DNA AE | Skin | Camaçari | Camaçari - Bahia | Canine | DEL |
| 51 | No | RUR 65 PELE | Skin | Camaçari | Camaçari - Bahia | Canine | DEL |
| 52 | Yes | RUR 376 PELE 3T | Skin | Camaçari | Camaçari - Bahia | Canine | No amplification detected |
| 53 | Yes | RUR 154 PELE | Skin | Camaçari | Camaçari - Bahia | Canine | Inconclusive |
| 54 | Yes | RUR 245 BACO 2T | Spleen | Camaçari | Camaçari - Bahia | Canine | No amplification detected |
| 55 | Yes | RUR 151 PELE | Skin | Camaçari | Camaçari - Bahia | Canine | DEL |
| 56 | Yes | RUR 384 PELE 3T | Skin | Camaçari | Camaçari - Bahia | Canine | DEL |
| 57 | Yes | RUR 231 BACO | Spleen | Camaçari | Camaçari - Bahia | Canine | DEL |
| 58 | Yes | RUR 343 BACO 3T | Spleen | Camaçari | Camaçari - Bahia | Canine | DEL |
| 59 | Yes | RUR 62 BACO 1T | Spleen | Camaçari | Camaçari - Bahia | Canine | No amplification detected |
| 60 | Yes | RUR 361 PELE 2T | Skin | Camaçari | Camaçari - Bahia | Canine | DEL |
| 61 | No | RUR 390 BACO 2T | Spleen | Camaçari | Camaçari - Bahia | Canine | DEL |
| 61 | No | RUR 390 PELE 3T | Skin | Camaçari | Camaçari - Bahia | Canine | DEL |
| 62 | No | RUR 110 PELE 3T | Skin | Camaçari | Camaçari - Bahia | Canine | DEL |
| 62 | No | RUR 110 PELE CT | Skin | Camaçari | Camaçari - Bahia | Canine | DEL |
| 63 | No | RUR 450 PELE 2T | Skin | Camaçari | Camaçari - Bahia | Canine | NonDEL |
| 63 | No | RUR 450 PELE 4T | Skin | Camaçari | Camaçari - Bahia | Canine | NonDEL |
| 63 | No | RUR 450 PELE 1T | Skin | Camaçari | Camaçari - Bahia | Canine | NonDEL |
| 63 | No | RUR 450 PELE 3T | Skin | Camaçari | Camaçari - Bahia | Canine | NonDEL |
| 64 | Yes | RUR 014 PELE 3T | Skin | Camaçari | Camaçari - Bahia | Canine | DEL |
| 65 | No | RUR 354 PELE 2T | Skin | Camaçari | Camaçari - Bahia | Canine | DEL |
| 65 | No | RUR 354 PELE | Skin | Camaçari | Camaçari - Bahia | Canine | DEL |
| 65 | No | RUR 354 PELE 1T | Skin | Camaçari | Camaçari - Bahia | Canine | DEL |
| 66 | No | RUR 001 PELE 2T | Skin | Camaçari | Camaçari - Bahia | Canine | DEL |
| 66 | No | RUR 001 PELE 3T | Skin | Camaçari | Camaçari - Bahia | Canine | DEL |
| 67 | No | RUR 374 PELE 3T | Skin | Camaçari | Camaçari - Bahia | Canine | DEL |
| 67 | No | RUR 374 PELE 4T | Skin | Camaçari | Camaçari - Bahia | Canine | DEL |
| 68 | Yes | RUR 063 DNA AE | Skin | Camaçari | Camaçari - Bahia | Canine | DEL |
| 69 | No | RUR 365 PELE 2T | Skin | Camaçari | Camaçari - Bahia | Canine | DEL |
| 69 | No | RUR 365 PELE 1T | Skin | Camaçari | Camaçari - Bahia | Canine | DEL |
| 69 | No | RUR 365 PELE 3T | Skin | Camaçari | Camaçari - Bahia | Canine | DEL |
| 70 | Yes | RUR 12 PELE | Skin | Camaçari | Camaçari - Bahia | Canine | DEL |
| 71 | Yes | RUR 449 PELE 2T | Skin | Camaçari | Camaçari - Bahia | Canine | DEL |
| 72 | Yes | RUR 275 PELE 1T | Skin | Camaçari | Camaçari - Bahia | Canine | DEL |
| 73 | Yes | RUR 298 PELE 3T | Skin | Camaçari | Camaçari - Bahia | Canine | DEL |
| 74 | Yes | RUR 127 PELE 3T | Skin | Camaçari | Camaçari - Bahia | Canine | DEL |
| 75 | No | RUR 093 PELE 2T | Skin | Camaçari | Camaçari - Bahia | Canine | DEL |
| 75 | No | RUR 093 PELE CT | Skin | Camaçari | Camaçari - Bahia | Canine | DEL |
| 76 | Yes | RUR 465 PELE 2T | Skin | Camaçari | Camaçari - Bahia | Canine | DEL |
| 77 | Yes | RUR 452 PELE | Skin | Camaçari | Camaçari - Bahia | Canine | DEL |
| 78 | Yes | RUR 452 PELE 2T | Skin | Camaçari | Camaçari - Bahia | Canine | DEL |
| 79 | Yes | RUR 066 PELE 2T | Skin | Camaçari | Camaçari - Bahia | Canine | DEL |
| 80 | Yes | RUR 464 PELE 1T | Skin | Camaçari | Camaçari - Bahia | Canine | DEL |
| 81 | Yes | RUR 4 DNA AE | Skin | Camaçari | Camaçari - Bahia | Canine | DEL |
| 82 | Yes | 516 | Skin | Mato Grosso do Sul | Mato Grosso do Sul | Coati | NonDEL |
| 83 | Yes | 333 | Skin | Mato Grosso do Sul | Mato Grosso do Sul | Coati | DEL |
| 84 | Yes | 349 | Skin | Mato Grosso do Sul | Mato Grosso do Sul | Coati | No amplification detected |
| 85 | No | 352 B | Skin | Mato Grosso do Sul | Mato Grosso do Sul | Coati | No amplification detected |
| 85 | No | 352 C | Skin | Mato Grosso do Sul | Mato Grosso do Sul | Coati | DEL |
| 86 | Yes | 401 | Skin | Mato Grosso do Sul | Mato Grosso do Sul | Coati | DEL |
| 87 | Yes | 403 | Skin | Mato Grosso do Sul | Mato Grosso do Sul | Coati | DEL |
| 88 | Yes | 420 | Skin | Mato Grosso do Sul | Mato Grosso do Sul | Coati | DEL |
| 89 | Yes | 513 | Skin | Mato Grosso do Sul | Mato Grosso do Sul | Coati | DEL |
| 90 | Yes | 514 | Skin | Mato Grosso do Sul | Mato Grosso do Sul | Coati | DEL |
| 91 | No | RUR 001 1T | Spleen | Camaçari | Camaçari - Bahia | Canine | DEL |
| 91 | No | RUR 001 2T | Spleen | Camaçari | Camaçari - Bahia | Canine | DEL |
| 91 | No | RUR 001 3T | Spleen | Camaçari | Camaçari - Bahia | Canine | DEL |
| 92 | No | RUR 002 2T | Spleen | Camaçari | Camaçari - Bahia | Canine | DEL |
| 92 | No | RUR 002 3T | Spleen | Camaçari | Camaçari - Bahia | Canine | DEL |
| 93 | No | RUR 004 1T | Spleen | Camaçari | Camaçari - Bahia | Canine | DEL |
| 93 | No | RUR 004 2T | Spleen | Camaçari | Camaçari - Bahia | Canine | DEL |
| 93 | No | RUR 004 3T | Spleen | Camaçari | Camaçari - Bahia | Canine | DEL |
| 93 | No | RUR 004 4T | Spleen | Camaçari | Camaçari - Bahia | Canine | DEL |
| 94 | No | RUR 012 | Spleen | Camaçari | Camaçari - Bahia | Canine | DEL |
| 94 | No | RUR 012 2T | Spleen | Camaçari | Camaçari - Bahia | Canine | DEL |
| 94 | No | RUR 012 3T | Spleen | Camaçari | Camaçari - Bahia | Canine | DEL |
| 95 | Yes | RUR 014 2T | Spleen | Camaçari | Camaçari - Bahia | Canine | DEL |
| 95 | Yes | RUR 014 3T | Spleen | Camaçari | Camaçari - Bahia | Canine | Inconclusive |
| 96 | No | RUR 018 1T | Spleen | Camaçari | Camaçari - Bahia | Canine | Inconclusive |
| 96 | No | RUR 018 2T | Spleen | Camaçari | Camaçari - Bahia | Canine | DEL |
| 96 | No | RUR 018 3T | Spleen | Camaçari | Camaçari - Bahia | Canine | DEL |
| 97 | No | RUR 066 1T | Spleen | Camaçari | Camaçari - Bahia | Canine | DEL |
| 97 | No | RUR 066 2T | Spleen | Camaçari | Camaçari - Bahia | Canine | DEL |
| 98 | No | RUR 073 1T | Spleen | Camaçari | Camaçari - Bahia | Canine | DEL |
| 98 | No | RUR 073 2T | Spleen | Camaçari | Camaçari - Bahia | Canine | DEL |
| 99 | Yes | RUR 074 1T | Spleen | Camaçari | Camaçari - Bahia | Canine | DEL |
| 100 | No | RUR 093 | Spleen | Camaçari | Camaçari - Bahia | Canine | DEL |
| 100 | No | RUR 093 1T | Spleen | Camaçari | Camaçari - Bahia | Canine | DEL |
| 100 | No | RUR 093 2T | Spleen | Camaçari | Camaçari - Bahia | Canine | DEL |
| 101 | Yes | RUR 094 | Spleen | Camaçari | Camaçari - Bahia | Canine | DEL |
| 102 | No | RUR 110 2T | Spleen | Camaçari | Camaçari - Bahia | Canine | Inconclusive |
| 102 | No | RUR 110 3T | Spleen | Camaçari | Camaçari - Bahia | Canine | Inconclusive |
| 102 | No | RUR 110 4T | Spleen | Camaçari | Camaçari - Bahia | Canine | DEL |
| 103 | No | RUR 127 1T | Spleen | Camaçari | Camaçari - Bahia | Canine | Inconclusive |
| 103 | No | RUR 127 2T | Spleen | Camaçari | Camaçari - Bahia | Canine | DEL |
| 103 | No | RUR 127 3T | Spleen | Camaçari | Camaçari - Bahia | Canine | DEL |
| 104 | Yes | RUR 218 4T | Spleen | Camaçari | Camaçari - Bahia | Canine | DEL |
| 105 | Yes | RUR 273 4T | Spleen | Camaçari | Camaçari - Bahia | Canine | Inconclusive |
| 106 | No | RUR 275 | Spleen | Camaçari | Camaçari - Bahia | Canine | DEL |
| 106 | No | RUR 275 1T | Spleen | Camaçari | Camaçari - Bahia | Canine | Inconclusive |
| 107 | No | RUR 298 | Spleen | Camaçari | Camaçari - Bahia | Canine | Inconclusive |
| 107 | No | RUR 298 1T | Spleen | Camaçari | Camaçari - Bahia | Canine | Inconclusive |
| 107 | No | RUR 298 2T | Spleen | Camaçari | Camaçari - Bahia | Canine | DEL |
| 107 | No | RUR 298 3T | Spleen | Camaçari | Camaçari - Bahia | Canine | Inconclusive |
| 108 | No | RUR 327 CT | Spleen | Camaçari | Camaçari - Bahia | Canine | Inconclusive |
| 108 | No | RUR 327 1T | Spleen | Camaçari | Camaçari - Bahia | Canine | Inconclusive |
| 109 | No | RUR 354 CT | Spleen | Camaçari | Camaçari - Bahia | Canine | DEL |
| 109 | No | RUR 354 1T | Spleen | Camaçari | Camaçari - Bahia | Canine | DEL |
| 109 | No | RUR 354 2T | Spleen | Camaçari | Camaçari - Bahia | Canine | DEL |
| 110 | No | RUR 365 CT | Spleen | Camaçari | Camaçari - Bahia | Canine | Inconclusive |
| 110 | No | RUR 365 1T | Spleen | Camaçari | Camaçari - Bahia | Canine | DEL |
| 110 | No | RUR 365 2T | Spleen | Camaçari | Camaçari - Bahia | Canine | DEL |
| 110 | No | RUR 365 3T | Spleen | Camaçari | Camaçari - Bahia | Canine | DEL |
| 111 | No | RUR 374 3T | Spleen | Camaçari | Camaçari - Bahia | Canine | DEL |
| 111 | No | RUR 374 4T | Spleen | Camaçari | Camaçari - Bahia | Canine | DEL |
| 112 | Yes | RUR 403 3T | Spleen | Camaçari | Camaçari - Bahia | Canine | Inconclusive |
| 113 | No | RUR 421 CT | Spleen | Camaçari | Camaçari - Bahia | Canine | DEL |
| 113 | No | RUR 421 1T | Spleen | Camaçari | Camaçari - Bahia | Canine | Inconclusive |
| 113 | No | RUR 421 2T | Spleen | Camaçari | Camaçari - Bahia | Canine | DEL |
| 114 | No | RUR 436 1T | Spleen | Camaçari | Camaçari - Bahia | Canine | Inconclusive |
| 114 | No | RUR 436 2T | Spleen | Camaçari | Camaçari - Bahia | Canine | DEL |
| 114 | No | RUR 436 4T | Spleen | Camaçari | Camaçari - Bahia | Canine | DEL |
| 115 | Yes | RUR 446 1T | Spleen | Camaçari | Camaçari - Bahia | Canine | Inconclusive |
| 116 | No | RUR 449 1T | Spleen | Camaçari | Camaçari - Bahia | Canine | DEL |
| 116 | No | RUR 449 2T | Spleen | Camaçari | Camaçari - Bahia | Canine | DEL |
| 117 | No | RUR 450 CT | Spleen | Camaçari | Camaçari - Bahia | Canine | NonDEL |
| 117 | No | RUR 450 1T | Spleen | Camaçari | Camaçari - Bahia | Canine | NonDEL |
| 117 | No | RUR 450 2T | Spleen | Camaçari | Camaçari - Bahia | Canine | NonDEL |
| 117 | No | RUR 450 3T | Spleen | Camaçari | Camaçari - Bahia | Canine | NonDEL |
| 117 | No | RUR 450 4T | Spleen | Camaçari | Camaçari - Bahia | Canine | NonDEL |
| 118 | No | RUR 452 CT | Spleen | Camaçari | Camaçari - Bahia | Canine | DEL |
| 118 | No | RUR 452 2T | Spleen | Camaçari | Camaçari - Bahia | Canine | DEL |
| 119 | No | RUR 465 CT | Spleen | Camaçari | Camaçari - Bahia | Canine | DEL |
| 119 | No | RUR 465 1T | Spleen | Camaçari | Camaçari - Bahia | Canine | DEL |
| 119 | No | RUR 465 2T | Spleen | Camaçari | Camaçari - Bahia | Canine | DEL |
| 120 | Yes | CAI7 | Limph node | Caieiras | Caieiras | Canine | DEL |
| 121 | Yes | CAI8 | Limph node | Caieiras | Caieiras | Canine | DEL |
| 122 | Yes | CAI9 | Limph node | Caieiras | Caieiras | Canine | DEL |
| 123 | Yes | CAI10 | Limph node | Caieiras | Caieiras | Canine | Inconclusive |
| 124 | Yes | CAI11 | Limph node | Caieiras | Caieiras | Canine | Inconclusive |
| 125 | Yes | CAI12 | Limph node | Caieiras | Caieiras | Canine | NonDEL |
| 126 | Yes | CAI13 | Limph node | Caieiras | Caieiras | Canine | Inconclusive |
| 127 | Yes | CAI14 | Limph node | Caieiras | Caieiras | Canine | Inconclusive |
| 128 | Yes | ATB1 | Limph node | Araçatuba | Araçatuba | Canine | NonDEL |
| 129 | Yes | ATB2 | Limph node | Araçatuba | Araçatuba | Canine | Inconclusive |
| 130 | Yes | ATB3 | Limph node | Araçatuba | Araçatuba | Canine | NonDEL |
| 131 | Yes | ATB4 | Limph node | Araçatuba | Araçatuba | Canine | Inconclusive |
| 132 | Yes | ATB5 | Limph node | Araçatuba | Araçatuba | Canine | Inconclusive |
| 133 | Yes | JAV1 | Limph node | Jales | Jales | Canine | NonDEL |
| 134 | Yes | JAV2 | Limph node | Jales | Jales | Canine | NonDEL |
| 135 | Yes | SF21 | Limph node | Santa Fé do Sul | Santa Fé do Sul | Canine | NonDEL |
| 136 | Yes | SF22 | Limph node | Santa Fé do Sul | Santa Fé do Sul | Canine | NonDEL |
| 137 | Yes | SF23 | Limph node | Santa Fé do Sul | Santa Fé do Sul | Canine | Inconclusive |
| 138 | Yes | TIP01 | Limph node | São Paulo |  | Canine | DEL |
| 139 | Yes | EMB1 | Limph node | Embu das Artes | Embú das Artes | Canine | Inconclusive |
| 140 | Yes | ESP1 | Limph node | São Paulo |  | Canine | DEL |
| 141 | Yes | ESP2 | Limph node | São Paulo |  | Canine | Inconclusive |
| 142 | Yes | ESP3 | Limph node | São Paulo |  | Canine | Inconclusive |
| 143 | Yes | ESP4 | Limph node | São Paulo |  | Canine | Inconclusive |
| 144 | Yes | ESP5 | Limph node | São Paulo |  | Canine | Inconclusive |
| 145 | Yes | ESP6 | Limph node | São Paulo |  | Canine | DEL |
| 146 | Yes | ESP7 | Limph node | São Paulo |  | Canine | Inconclusive |
| 147 | Yes | ESP9 | Limph node | São Paulo |  | Canine | Inconclusive |
| 148 | Yes | ESP10 | Limph node | São Paulo |  | Canine | Inconclusive |
| 149 | Yes | 1 | Limph node | São Paulo |  | Canine | No amplification detected |
| 150 | Yes | 2 | Limph node | São Paulo |  | Canine | No amplification detected |
| 151 | Yes | 3 | Limph node | São Paulo |  | Canine | No amplification detected |
| 152 | Yes | 4 | Limph node | São Paulo |  | Canine | No amplification detected |
| 153 | Yes | 5 | Limph node | São Paulo |  | Canine | No amplification detected |
| 154 | Yes | 6 | Limph node | São Paulo |  | Canine | No amplification detected |
| 155 | Yes | 7 | Limph node | São Paulo |  | Canine | No amplification detected |
| 156 | Yes | 8 | Limph node | São Paulo |  | Canine | No amplification detected |
| 157 | Yes | 9 | Limph node | São Paulo |  | Canine | NonDEL |
| 158 | Yes | 10 | Limph node | São Paulo |  | Canine | No amplification detected |
| 159 | Yes | 11 | Limph node | São Paulo |  | Canine | No amplification detected |
| 160 | Yes | 12 | Limph node | São Paulo |  | Canine | No amplification detected |
| 161 | Yes | 13 | Limph node | São Paulo |  | Canine | No amplification detected |
| 162 | Yes | 14 | Limph node | São Paulo |  | Canine | No amplification detected |
| 163 | Yes | 15 | Limph node | São Paulo |  | Canine | No amplification detected |
| 164 | Yes | 16 | Limph node | São Paulo |  | Canine | No amplification detected |
| 165 | Yes | 17 | Limph node | São Paulo |  | Canine | No amplification detected |
| 166 | Yes | 18 | Limph node | São Paulo |  | Canine | No amplification detected |
| 167 | Yes | 19 | Limph node | São Paulo |  | Canine | No amplification detected |
| 168 | Yes | 20 | Limph node | São Paulo |  | Canine | No amplification detected |
| 169 | Yes | 188 | Limph node | São Paulo |  | Canine | MIX qPCRprofile |
| 170 | Yes | 195 | Limph node | São Paulo |  | Canine | MIX qPCRprofile |
| 171 | Yes | 201 | Limph node | São Paulo |  | Canine | NonDEL |
| 172 | Yes | 208 | Limph node | São Paulo |  | Canine | NonDEL |
| 173 | Yes | 215 | Limph node | São Paulo |  | Canine | No amplification detected |
| 174 | Yes | 221 | Limph node | São Paulo |  | Canine | Inconclusive |
| 175 | Yes | 227 | Limph node | São Paulo |  | Canine | MIX qPCRprofile |
| 176 | Yes | 233 | Limph node | São Paulo |  | Canine | MIX qPCRprofile |
| 177 | Yes | 239 | Limph node | São Paulo |  | Canine | No amplification detected |
| 178 | Yes | VOTU1 | Limph node | Votuporanga | Votuporanga | Canine | Inconclusive |
| 179 | Yes | VOTU11 | Limph node | Votuporanga | Votuporanga | Canine | DEL |
| 180 | Yes | VOTU14 | Limph node | Votuporanga | Votuporanga | Canine | Inconclusive |
| 181 | Yes | VOTU22 | Limph node | Votuporanga | Votuporanga | Canine | Inconclusive |
| 182 | Yes | VOTU23 | Limph node | Votuporanga | Votuporanga | Canine | MIX qPCRprofile |
| 183 | Yes | VOTU25 | Limph node | Votuporanga | Votuporanga | Canine | DEL |
| 184 | Yes | VOTU26 | Limph node | Votuporanga | Votuporanga | Canine | Inconclusive |
| 185 | Yes | VOTU27 | Limph node | Votuporanga | Votuporanga | Canine | MIX qPCRprofile |
| 186 | Yes | VOTU28 | Limph node | Votuporanga | Votuporanga | Canine | DEL |
| 187 | Yes | VOTU29 | Limph node | Votuporanga | Votuporanga | Canine | NonDEL |
| 188 | Yes | VOTU30 | Limph node | Votuporanga | Votuporanga | Canine | DEL |
| 189 | Yes | VOTU31 | Limph node | Votuporanga | Votuporanga | Canine | NonDEL |
| 190 | Yes | VOTU32 | Limph node | Votuporanga | Votuporanga | Canine | NonDEL |
| 191 | Yes | VOTU42 | Limph node | Votuporanga | Votuporanga | Canine | DEL |
| 192 | Yes | VOTU43 | Limph node | Votuporanga | Votuporanga | Canine | NonDEL |
| 193 | Yes | VOTU44 | Limph node | Votuporanga | Votuporanga | Canine | MIX qPCR profile |
| 194 | Yes | VOTU45 | Limph node | Votuporanga | Votuporanga | Canine | NonDEL |
| 195 | Yes | VOTU46 | Limph node | Votuporanga | Votuporanga | Canine | Inconclusive |
| 196 | Yes | VOTU47 | Limph node | Votuporanga | Votuporanga | Canine | NonDEL |
| 197 | Yes | VOTU48 | Limph node | Votuporanga | Votuporanga | Canine | Inconclusive |
| 198 | Yes | VOTU49 | Limph node | Votuporanga | Votuporanga | Canine | Inconclusive |
| 199 | Yes | VOTU52 | Limph node | Votuporanga | Votuporanga | Canine | Inconclusive |
| 200 | Yes | VOTU53 | Limph node | Votuporanga | Votuporanga | Canine | NonDEL |
| 201 | Yes | VOTU54 | Limph node | Votuporanga | Votuporanga | Canine | Inconclusive |
| 202 | Yes | VOTU55 | Limph node | Votuporanga | Votuporanga | Canine | MIX qPCR profile |
| 203 | Yes | VOTU56 | Limph node | Votuporanga | Votuporanga | Canine | Inconclusive |
| 204 | Yes | VOTU68 | Limph node | Votuporanga | Votuporanga | Canine | Inconclusive |
| 205 | Yes | VOTU70 | Limph node | Votuporanga | Votuporanga | Canine | MIX qPCR profile |
| 206 | Yes | VOTU71 | Limph node | Votuporanga | Votuporanga | Canine | NonDEL |
| 207 | Yes | VOTU72 | Limph node | Votuporanga | Votuporanga | Canine | NonDEL |
| 208 | Yes | VOTU73 | Limph node | Votuporanga | Votuporanga | Canine | NonDEL |
| 209 | Yes | VOTU74 | Limph node | Votuporanga | Votuporanga | Canine | MIX qPCR profile |
| 210 | Yes | VOTU75 | Limph node | Votuporanga | Votuporanga | Canine | Inconclusive |
| 211 | Yes | VOTU88 | Limph node | Votuporanga | Votuporanga | Canine | MIX qPCR profile |
| 212 | Yes | FER2 | Limph node | Fernandópolis | Fernandópolis | Canine | NonDEL |
| 213 | Yes | FER5 | Limph node | Fernandópolis | Fernandópolis | Canine | Inconclusive |
| 214 | Yes | FER10 | Limph node | Fernandópolis | Fernandópolis | Canine | Inconclusive |
| 215 | Yes | FER12 | Limph node | Fernandópolis | Fernandópolis | Canine | NonDEL |
| 216 | Yes | FER13 | Limph node | Fernandópolis | Fernandópolis | Canine | NonDEL |
| 217 | Yes | FER14 | Limph node | Fernandópolis | Fernandópolis | Canine | DEL |
| 218 | Yes | FER15 | Limph node | Fernandópolis | Fernandópolis | Canine | DEL |
| 219 | Yes | FER16 | Limph node | Fernandópolis | Fernandópolis | Canine | DEL |
| 220 | Yes | FER18 | Limph node | Fernandópolis | Fernandópolis | Canine | Inconclusive |
| 221 | Yes | FER19 | Limph node | Fernandópolis | Fernandópolis | Canine | DEL |
| 222 | Yes | FER21 | Limph node | Fernandópolis | Fernandópolis | Canine | DEL |
| 223 | Yes | FER22 | Limph node | Fernandópolis | Fernandópolis | Canine | DEL |
| 224 | Yes | FER24 | Limph node | Fernandópolis | Fernandópolis | Canine | DEL |
| 225 | Yes | FER25 | Limph node | Fernandópolis | Fernandópolis | Canine | DEL |
| 226 | Yes | FER27 | Limph node | Fernandópolis | Fernandópolis | Canine | MIX qPCR profile |
| 227 | Yes | FER28 | Limph node | Fernandópolis | Fernandópolis | Canine | DEL |
| 228 | Yes | BAU09 | Limph node | Bauru | Bauru | Canine | NonDEL |
| 229 | Yes | BAU06 | Limph node | Bauru | Bauru | Canine | DEL |
| 230 | Yes | BAU10 | Limph node | Bauru | Bauru | Canine | NonDEL |
| 231 | Yes | BAU49 | Limph node | Bauru | Bauru | Canine | DEL |
| 232 | Yes | BAU48 | Limph node | Bauru | Bauru | Canine | MIX qPCR profile |
| 233 | Yes | BAU62 | Limph node | Bauru | Bauru | Canine | NonDEL |
| 234 | Yes | BAU23 | Limph node | Bauru | Bauru | Canine | NonDEL |
| 235 | Yes | BAU16 | Limph node | Bauru | Bauru | Canine | NonDEL |
| 236 | Yes | BAU65 | Limph node | Bauru | Bauru | Canine | DEL |
| 237 | Yes | BAU50 | Limph node | Bauru | Bauru | Canine | DEL |
| 238 | Yes | BAU37 | Limph node | Bauru | Bauru | Canine | DEL |
| 239 | Yes | BAU38 | Limph node | Bauru | Bauru | Canine | DEL |
| 240 | Yes | BAU05 | Limph node | Bauru | Bauru | Canine | NonDEL |
| 241 | Yes | BAU15 | Limph node | Bauru | Bauru | Canine | DEL |
| 242 | Yes | BAU46 | Limph node | Bauru | Bauru | Canine | DEL |
| 243 | Yes | BAU21 | Limph node | Bauru | Bauru | Canine | DEL |
| 244 | Yes | BAU58 | Limph node | Bauru | Bauru | Canine | NonDEL |
| 245 | Yes | BAU26 | Limph node | Bauru | Bauru | Canine | DEL |
| 246 | Yes | BAU79 | Limph node | Bauru | Bauru | Canine | NonDEL |
| 247 | Yes | BAU66 | Limph node | Bauru | Bauru | Canine | DEL |
| 248 | Yes | BAU07 | Limph node | Bauru | Bauru | Canine | DEL |
| 249 | Yes | BAU03 | Limph node | Bauru | Bauru | Canine | NonDEL |
| 250 | Yes | BAU02 | Limph node | Bauru | Bauru | Canine | DEL |
| 251 | Yes | BAU04 | Limph node | Bauru | Bauru | Canine | NonDEL |
| 252 | Yes | BAU01 | Limph node | Bauru | Bauru | Canine | DEL |
| 253 | Yes | BAU20 | Limph node | Bauru | Bauru | Canine | NonDEL |
| 254 | Yes | BAU19 | Limph node | Bauru | Bauru | Canine | NonDEL |
| 255 | Yes | BAU08 | Limph node | Bauru | Bauru | Canine | DEL |
| 256 | Yes | BAU22 | Limph node | Bauru | Bauru | Canine | NonDEL |
| 257 | Yes | BAU28 | Limph node | Bauru | Bauru | Canine | NonDEL |
| 258 | Yes | BAU56 | Limph node | Bauru | Bauru | Canine | DEL |
| 259 | Yes | BAU60 | Limph node | Bauru | Bauru | Canine | DEL |
| 260 | Yes | BAU72 | Limph node | Bauru | Bauru | Canine | NonDEL |
| 261 | Yes | BAU69 | Limph node | Bauru | Bauru | Canine | DEL |
| 262 | Yes | BAU55 | Limph node | Bauru | Bauru | Canine | DEL |
| 263 | Yes | gPL18 | Skin | Uruguay | Uruguai | Canine | DEL |
| 264 | Yes | bPL7 | Skin | Uruguay | Uruguai | Canine | DEL |
| 265 | Yes | gCH2 | Skin | Uruguay | Uruguai | Canine | DEL |
| 266 | Yes | bCH11 | Skin | Uruguay | Uruguai | Canine | DEL |
| 267 | Yes | mCO2 | Skin | Uruguay | Uruguai | Canine | DEL |
| 268 | Yes | 1 47-P | Skin | Mato Grosso do Sul | Mato Grosso do Sul | Canine | Inconclusive |
| 269 | No | 3. 49-P | Skin | Mato Grosso do Sul | Mato Grosso do Sul | Canine | Inconclusive |
| 269 | No | 4. 49-MO | Bone marrow | Mato Grosso do Sul | Mato Grosso do Sul | Canine | Inconclusive |
| 270 | Yes | 5. 50-P | Skin | Mato Grosso do Sul | Mato Grosso do Sul | Canine | Inconclusive |
| 271 | No | 6. 51-P | Skin | Mato Grosso do Sul | Mato Grosso do Sul | Canine | Inconclusive |
| 271 | No | 7. 51-MO | Bone marrow | Mato Grosso do Sul | Mato Grosso do Sul | Canine | Inconclusive |
| 272 | Yes | 8. 52-P | Skin | Mato Grosso do Sul | Mato Grosso do Sul | Canine | Inconclusive |
| 273 | No | 9. 53-P | Skin | Mato Grosso do Sul | Mato Grosso do Sul | Canine | DEL |
| 273 | No | 10. 53-MO | Bone marrow | Mato Grosso do Sul | Mato Grosso do Sul | Canine | DEL |
| 274 | No | 11. 54-P | Skin | Mato Grosso do Sul | Mato Grosso do Sul | Canine | Inconclusive |
| 274 | No | 12. 54-MO | Bone marrow | Mato Grosso do Sul | Mato Grosso do Sul | Canine | Inconclusive |
| 275 | No | 13. RP1-P | Skin | Mato Grosso do Sul | Mato Grosso do Sul | Canine | Inconclusive |
| 275 | No | 14. RP1-MO | Bone marrow | Mato Grosso do Sul | Mato Grosso do Sul | Canine | Inconclusive |
| 276 | No | 17. RP3-P | Skin | Mato Grosso do Sul | Mato Grosso do Sul | Canine | DEL |
| 276 | No | 18. RP3-MO | Bone marrow | Mato Grosso do Sul | Mato Grosso do Sul | Canine | DEL |
| 276 | No | 19. RP3-LF | Limph node | Mato Grosso do Sul | Mato Grosso do Sul | Canine | DEL |
| 276 | No | 20. RP3-Linf. | Limph node | Mato Grosso do Sul | Mato Grosso do Sul | Canine | DEL |
| 277 | No | 21. RP4-P | Skin | Mato Grosso do Sul | Mato Grosso do Sul | Canine | DEL |
| 277 | No | 22. RP4-MO | Bone marrow | Mato Grosso do Sul | Mato Grosso do Sul | Canine | Inconclusive |
| 277 | No | 23. RP4-Linf. | Limph node | Mato Grosso do Sul | Mato Grosso do Sul | Canine | Inconclusive |
| 278 | No | 24. RP5-P | Skin | Mato Grosso do Sul | Mato Grosso do Sul | Canine | Inconclusive |
| 278 | No | 25. RP5-Linf | Limph node | Mato Grosso do Sul | Mato Grosso do Sul | Canine | Inconclusive |
| 279 | Yes | 2. 48-P | Skin | Mato Grosso do Sul | Mato Grosso do Sul | Canine | No amplification detected |
| 280 | No | 15. RP2-P | Skin | Mato Grosso do Sul | Mato Grosso do Sul | Canine | No amplification detected |
| 280 | No | 16. RP2-MO | Bone marrow | Mato Grosso do Sul | Mato Grosso do Sul | Canine | No amplification detected |
| 281 | Yes | B28 | Insect | Bolivia | Bolívia | Phlebotomine | DEL |
| 282 | Yes | B20 | Insect | Bolivia | Bolívia | Phlebotomine | DEL |
| 283 | Yes | B05 | Insect | Bolivia | Bolívia | Phlebotomine | No amplification detected |
| 284 | Yes | Princesa 152 - EX.1 LF | Limph node | Bauru | Bauru | Canine | NonDEL |
| 285 | Yes | Cristal 5682 - EX.1 LF | Limph node | Bauru | Bauru | Canine | NonDEL |
| 286 | Yes | Sheik 855 - EX.2 LF | Limph node | Bauru | Bauru | Canine | NonDEL |
| 287 | Yes | Mel 5645 - EX.1 LF | Limph node | Bauru | Bauru | Canine | NonDEL |
| 288 | Yes | Princesa 1155 - EX.2 LF | Limph node | Bauru | Bauru | Canine | NonDEL |
| 289 | Yes | Athena 5884 - EX.2 LF | Limph node | Bauru | Bauru | Canine | NonDEL |
| 290 | Yes | Jerry 4931 - EX.1 LF | Limph node | Bauru | Bauru | Canine | NonDEL |
| 291 | Yes | Zeus 3205 - EX.1 LF | Limph node | Bauru | Bauru | Canine | NonDEL |
| 292 | Yes | Nina 4840 - EX.1 LF | Limph node | Bauru | Bauru | Canine | NonDEL |
